# Supplementary material for: Universal patterns in egocentric communication networks
Source: Nat Commun. 2023 Aug 26;14:5217. doi: 10.1038/s41467-023-40888-5 (PMC10460427; doi:10.1038/s41467-023-40888-5)
Supplement: Supplementary file 1 — Supplementary Information [file 41467_2023_40888_MOESM1_ESM.pdf]

Supplementary Information for

# Universal patterns in egocentric communication networks

G. Iñiguez*, S. Heydari, J. Kertész, J. Saramäki*

*Corresponding author email: iniguezg@ceu.edu, saramaki@aalto.fi

## Contents

**S1 Communication data** **1**
S1.1 List of datasets . . . . . . . . . . 2
S1.1.1 Data acknowledgments . . . . . . . . . . 6
S1.2 Ego network properties, activity dispersion and connection kernel . . . . . . . . . . 6

**S2 Model of alter activity** **10**
S2.1 Master equation for activity dynamics . . . . . . . . . . 10
S2.1.1 Heterogeneous regime ($\beta > 1$): Alter activity is gamma-distributed . . . . . . . . . . 13
S2.1.2 Homogeneous regime ($\beta < 1$): Alter activity is Poisson-distributed . . . . . . . . . . 13

**S3 Fitting data and model** **16**
S3.1 Derivation of maximum likelihood estimates . . . . . . . . . . 16
S3.2 Goodness-of-fit test . . . . . . . . . . 17
S3.3 Activity regimes and persistence analysis in communication data . . . . . . . . . . 21

## S1 Communication data

We analyze several datasets of social interactions between individuals from a wide range of studies in the temporal networks literature (Table S1 and Fig. S1). Each dataset includes a time-ordered set of communication events between anonymized individuals $i$ and $j$ (according to hashed timestamps). For each dataset, we construct temporal ego networks for each individual so that the network for ego $i$ contains all events where $i$ participates. Therefore, each event connecting nodes $i$ and $j$ appears both in the ego network where $j$ is an alter of ego $i$, and in the ego network where $i$ is an alter of ego $j$ (except otherwise explicitly stated in Section S1.1). Table S1 lists basic properties of all datasets considered, starting with the system size $N_u$ (unfiltered number of egos) and number of events $V$ (all distinct contact events between egos and alters). We only consider egos with any level of heterogeneous alter activity, i.e. with mean alter activity $t$ larger than the minimum across its alters ($t > a_0$), leading to a reduced system size $N$ (filtered number of egos). Table S1 includes several properties of the filtered datasets: average degree $\langle k \rangle$ (mean number of alters per ego), average strength $\langle \tau \rangle$ (mean number of events per ego), average mean alter activity $\langle t \rangle$ (mean number of events per alter per ego), and average minimum/maximum alter activity $\langle a_0 \rangle$ and $\langle a_m \rangle$ (mean of lowest/highest alter activity per ego). We briefly describe below each dataset considered, including references to detailed studies and locations of publicly available data.

| Dataset | Event | $N_u$ | $V$ | $N$ | $\langle k \rangle$ | $\langle \tau \rangle$ | $\langle t \rangle$ | $\langle a_0 \rangle$ | $\langle a_m \rangle$ |
|---|---|---|---|---|---|---|---|---|---|
| Mobile (call) [1–7] | Phone calls | 5994967 | 1342862618 | 5431921 | 38.84 | 246.29 | 5.91 | 1.02 | 69.20 |
| Mobile (sms) [1–7] | Short messages | 5387745 | 613751054 | 4233187 | 16.95 | 143.30 | 7.49 | 1.15 | 60.68 |
| Mobile (Wu 1) [8] | Short messages | 44090 | 544817 | 16050 | 4.55 | 52.93 | 12.74 | 1.84 | 38.10 |
| Mobile (Wu 2) [8] | Short messages | 71042 | 636629 | 20534 | 4.71 | 43.86 | 10.66 | 1.91 | 29.86 |
| Mobile (Wu 3) [8] | Short messages | 14273 | 140611 | 4215 | 6.27 | 52.72 | 10.66 | 1.79 | 33.29 |
| Email (Enron) [9,10] | Emails | 86978 | 1134990 | 21984 | 22.52 | 96.43 | 3.26 | 1.15 | 16.27 |
| Email (Kiel) [11,12] | Emails | 57189 | 431864 | 9842 | 13.05 | 65.68 | 5.99 | 1.79 | 25.86 |
| Email (Uni) [12,13] | Emails | 3188 | 308730 | 2456 | 25.49 | 250.10 | 9.14 | 1.12 | 61.18 |
| Email (EU) [14,15] | Emails | 986 | 332334 | 866 | 36.92 | 766.98 | 18.22 | 1.08 | 194.64 |
| Facebook [12,16] | Online messages | 45813 | 854612 | 31429 | 11.04 | 53.22 | 4.08 | 1.17 | 17.07 |
| Messages [12,17,18] | Online messages | 35623 | 478015 | 20252 | 8.37 | 45.14 | 3.84 | 1.23 | 17.85 |
| Dating [12,19] | Online messages | 28972 | 430826 | 16239 | 13.05 | 51.44 | 3.44 | 1.11 | 13.29 |
| Forum [12,17,18] | Online messages | 7084 | 1428493 | 4122 | 65.22 | 691.15 | 2.83 | 1.01 | 57.41 |
| College [20,21] | Online messages | 1899 | 59835 | 1303 | 20.48 | 90.90 | 3.62 | 1.07 | 17.22 |
| CNS (call) [22,23] | Phone calls | 525 | 3234 | 285 | 3.25 | 19.00 | 6.25 | 1.76 | 12.47 |
| CNS (sms) [22,23] | Short messages | 568 | 24333 | 347 | 3.36 | 114.73 | 33.89 | 5.66 | 86.50 |

**Table S1. Datasets used in this study**. Characteristics of the available datasets, starting with system size $N_u$ (unfiltered number of egos) and number of events $V$ (all communication events between egos and alters). We only consider egos with mean alter activity larger than its minimum ($t > a_0$), leading to a system of size $N$ (filtered number of egos) with the following properties: average degree $\langle k \rangle$ (mean number of alters per ego), average strength $\langle \tau \rangle$ (mean number of events per ego), average mean alter activity $\langle t \rangle$ (mean number of events per alter per ego), and average minimum/maximum alter activity $\langle a_0 \rangle$ and $\langle a_m \rangle$ (mean of lowest/highest alter activity per ego). We include references to detailed studies of each dataset and locations of publicly available data.

## S1.1 List of datasets

**Phone calls (Mobile call & sms).** Mobile phone records dataset consisting of time-stamped communication logs between anonymized users, originally introduced in [1]. Data covers logs on outgoing communication of approximately 20% of the population of an undisclosed European country and spans a 6-months period in 2007. We have filtered out self-communication events (e.g., users messaging themselves) and records made by people under so-called 'family contracts' with the operator company (indicating that several individuals might have used the same phone line). After filtering, the dataset includes more than 5 million users, 1.3 billion calls, and 613 million short messages. Unlike the rest of the datasets below, this data is directional, meaning ego networks consist only of outgoing communication events. Data is not publicly available, but has been extensively studied in the literature (see, for example, [1–7]).

**Short messages (Wu 1, 2 & 3).** Dataset from a mobile phone operator including three charging accountant bills from three companies (denoted 1, 2, and 3) over a 1-month period. Each event comprises a sender mobile phone number, a recipient mobile phone number (both anonymized), and a hashed timestamp with a precision of 1 second [8]. Data is publicly available in the Supplementary Information of [8].

**Emails (Enron).** Dataset of email communication from the Enron corporation during 1999–2003, which was made public as a result of legal action by the Federal Energy Regulatory Commission in the US. A subset of the corpus including 200,399 messages sent between 158 users was originally studied in 2004 [9]. In 2015 this corpus was corrected and published in raw form [24]. Data we use comes from the Koblenz network collection [10] and corresponds to 1,148,072 emails between 87,273 addresses,

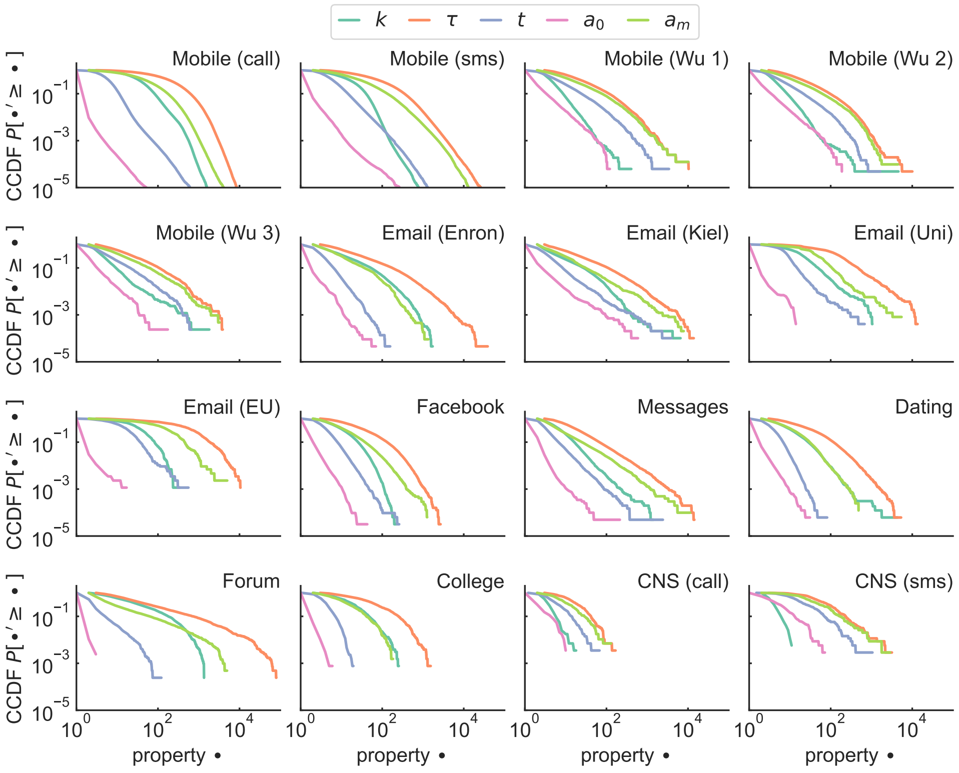


**Figure S1. Basic properties of communication datasets.** Complementary cumulative distribution functions (CCDFs) $P[\bullet' \geq \bullet]$ for several properties $\bullet$ of ego networks in each dataset: degree $k$ (number of alters of an ego), strength $\tau$ (number of events involving an ego), mean alter activity $t$ (average number of events per alter of an ego), minimum activity $a_0$ (minimum number of events with the same alter), and maximum activity $a_m$ (maximum number of events with the same alter). All properties are heterogeneously distributed across egos and alters, with some differences between datasets.

both inside and outside Enron. After filtering out events with equal sender and recipient, we obtain the slightly lower values of $N_u$ and $V$ in Table S1. Data is publicly available at `http://konect.cc/networks/enron/`.

**Emails (Kiel).** Dataset of log files of email server at Kiel University, recording source and destination of every email from or to a student account over a period of 112 days [11]. Data has also been analyzed in terms of temporal greedy walks in [12] (see Section S1.1.1 for data acknowledgments).

**Emails (Uni).** Dataset of log files of one of the main mail servers at an unnamed university, comprising email messages sent during a period of 83 days and connecting $\sim$ 10,000 users [13]. Data was reduced to the internal mail within the institution, leaving a set of 3,188 users interchanging 309,125 messages. The dataset has also been analyzed in terms of temporal greedy walks in [12]. The value of $V$ in [13] slightly differs when calculated directly from available data (see Table S1 and Section S1.1.1 for data acknowledgments).

**Emails (EU).** Dataset of email communication in a large European research institution from October 2003 to May 2005, comprising 3,038,531 messages between 287,755 addreses [14,15]. After focusing only on institution members and the emails sent between them, values of $N_u$ and $V$ decrease to those in

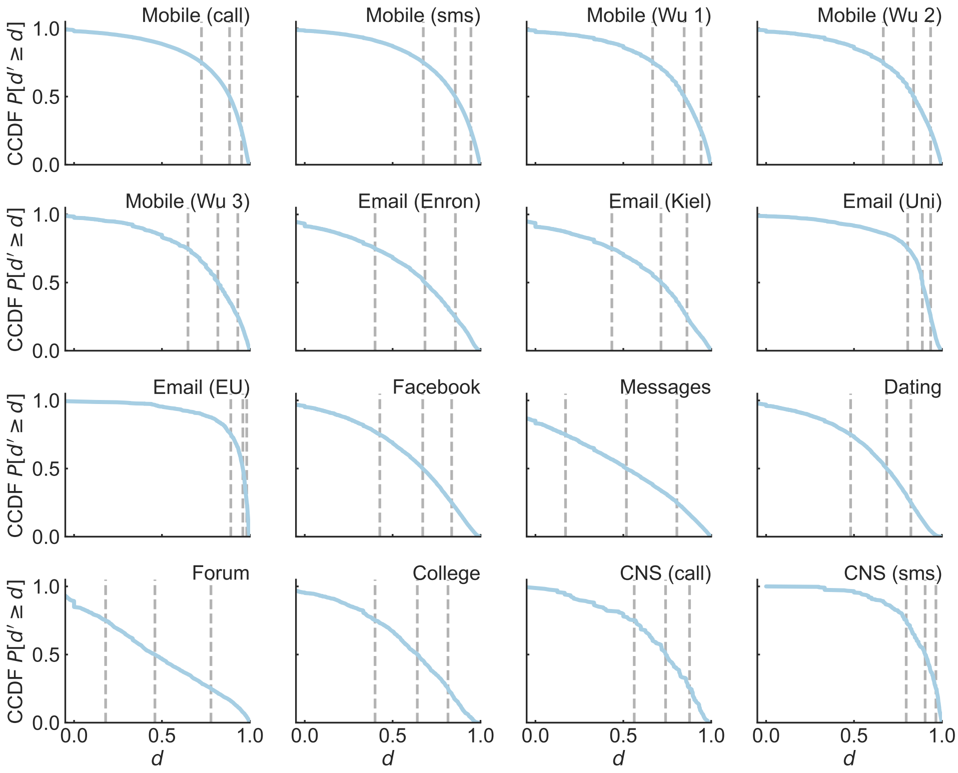


**Figure S2. Dispersion in communication activity.** Complementary cumulative distribution function (CCDF) $P[d' \geq d]$ of the number of egos having at least dispersion index $d$ in all considered datasets (calculated only for ego networks with more than 10 events, i.e. $\tau > 10$). Dashed lines indicate limits of the quartile ranges of the dispersion distribution $p_d$. Communication channels show broad variation in how egos allocate activity among alters.

Table S1. Data is publicly available at the SNAP repository in `https://snap.stanford.edu/data/email-Eu-core-temporal.html`.

**Online messages (Facebook).** Dataset on both friendship relationships and interactions for a large subset of the Facebook New Orleans social network, comprising over 60,000 anonymized users and over 800,000 logged interactions (wall posts) between users in a period of two years [16]. Facebook links were crawled during December 29th, 2008 and January 3rd, 2009, starting from a single user and visiting friends with a breadth-first-search algorithm. Wall posts were then crawled between January 20th, 2009 and January 22nd, 2009 for all previously detected users. Wall post data spans from September 26th, 2006 to January 22nd, 2009. The dataset has also been analyzed in terms of temporal greedy walks in [12]. Data is publicly available at: `http://socialnetworks.mpi-sws.org/data-wosn2009.html`. Values of $N_u$ and $V$ in [16] differ when calculated directly from available data (see Table S1).

**Online messages (Messages & Forum).** Dataset from the social movie recommendation community Filmtipset (Sweden's largest and available since 2000), consisting of time-stamped communications (contact events) between 36,492 users during 7 years [18]. Available data corresponds to a user-to-user messaging channel where each user can send text messages to another user privately and only one user at a time (Messages), and an open forum where users comment on posts of other users, as many as are willing to participate (Forum). The dataset was originally studied in [17], and has also been analyzed in terms of temporal greedy walks in [12] (see Section S1.1.1 for data acknowledgments).

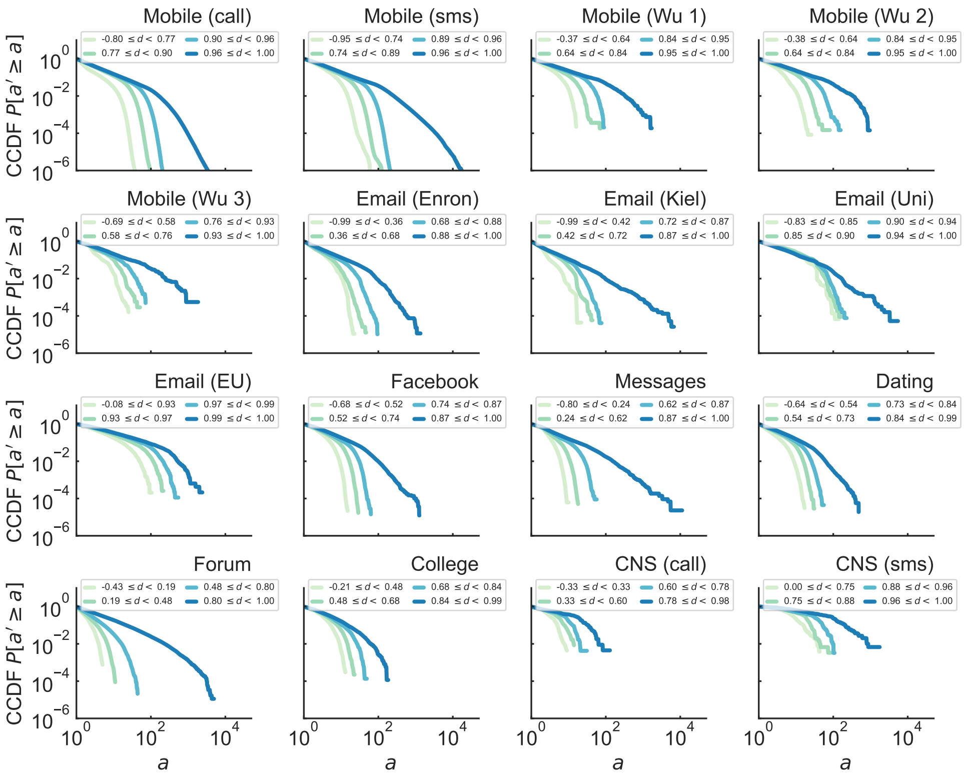


**Figure S3. Alter activity and dispersion index.** Complementary cumulative distribution function (CCDF) $P[a' \geq a]$ of the number of alters having at least activity $a$, restricted to ego networks in each quartile range of the distribution $p_d$ of the dispersion index $d$ (see Fig. S2), for all considered datasets. We only consider ego networks with $k \geq 10$, or $k \geq 2$ for CNS (call, sms). Egos with larger $d$ have a broader activity distribution $p_a$ [in accordance with Eq. (S1)], but also higher alter activities overall.

**Online messages (Dating).** Dataset from pussokram.com, a Swedish online community primarily intended for romantic communication and targeted at adolescents and young adults, consisting of all activity during 512 days from 13 February 2001 to 10 July 2002 among roughly 30,000 users [19]. Time-stamped contact events between users follow 4 modes of communication: private intra-community emails, guest book signing, friendship requests ('flirts'), and friendships. Data has also been analyzed in terms of temporal greedy walks in [12] (see Section S1.1.1 for data acknowledgments).

**Online messages (College).** Dataset of private messages sent on a Facebook-like online social network for students at the University of California, Irvine, from April to October 2004, where users could search the network for others and then initiate conversations based on their profile information [20, 21]. Data includes the 1,899 students that sent or received at least one message on the site, comprising 59,835 online messages over 20,296 directed ties between these users. The dataset is hosted by Tore Opsahl at `https://toreopsahl.com/datasets/#online_social_network` and is also publicly available from the SNAP repository at `https://snap.stanford.edu/data/CollegeMsg.html`.

**Copenhagen Networks Study (CNS call & sms)** Dataset of multi-channel, phone-enabled social interactions from the Copenhagen Networks Study (CNS) [22, 23]. The original study includes activity of roughly 1,000 individuals during 2012-2013 via Bluetooth interactions, calls, and messages [22]. Data used here is a selected portion of the full dataset as described in [23]. The selected dataset includes call

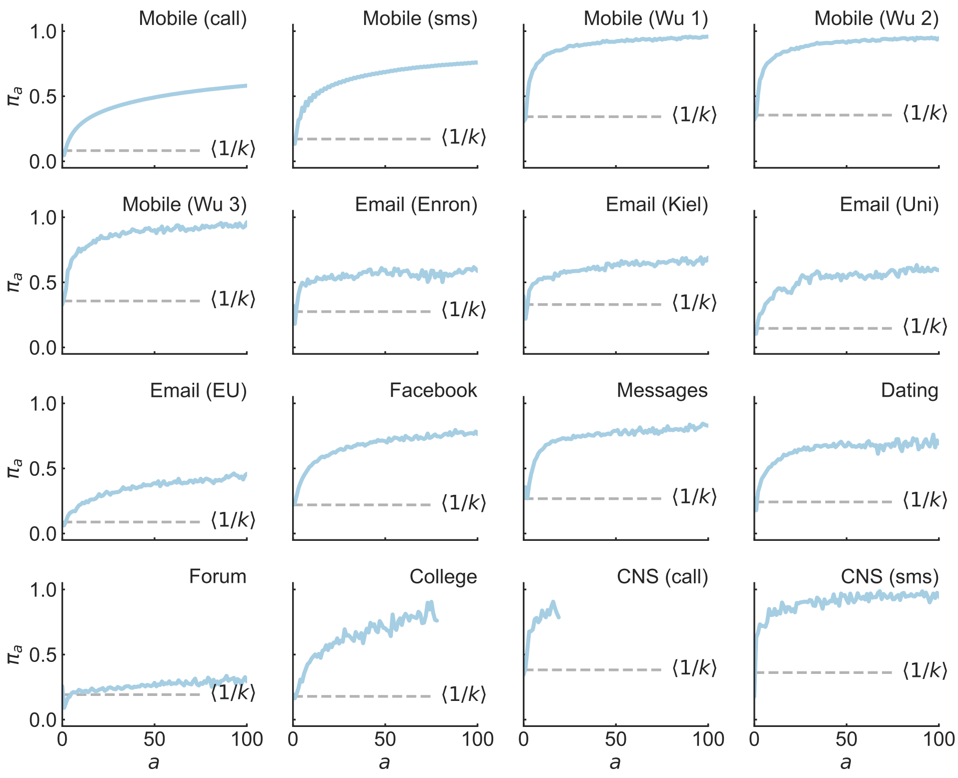


**Figure S4. Connection kernel in communication.** Probability $\pi_a$ that an alter with current activity $a$ communicates once more with the ego (averaged over time and subsets of at least 50 egos with degree $k \geq 2$ for each $a$ value), shown here for all considered datasets. The dashed line corresponds to the average baseline $\pi_a = \langle 1/k \rangle$ when communication events are distributed randomly. The growth of the connection kernel $\pi_a$ with activity indicates cumulative advantage, where alters with high prior activity receive more communication.

and short message logs between individuals, with data on timestamps of the call/message, anonymized user IDs, and call duration. We disregard missed calls, making the dataset smaller from the one in [23]. Data is publicly available via *figshare* in [25].

### S1.1.1 Data acknowledgments

We are grateful for data provision to Albert-László Barabási (Mobile call & sms) and Petter Holme (Email Kiel & Uni, Forum, Messages, Dating). We acknowledge the Department of Computer Science of Aalto University for access to processed versions of the non-public datasets used here (Mobile call & sms, Email Kiel & Uni, Forum, Messages, Dating).

## S1.2 Ego network properties, activity dispersion and connection kernel

In all considered datasets of communication, ego networks have heterogeneous structures and patterns of activity. For each ego network we measure the degree $k$, strength $\tau$, mean alter activity $t$, minimum alter activity $a_0$, and maximum alter activity $a_m$, and then see how these measures vary across egos. All properties show broad tails in their corresponding complementary cumulative distribution functions (CCDFs), the probability $P[\bullet' \geq \bullet]$ that an ego has property $\bullet'$ larger than a given value $\bullet$ (see Fig. S1).

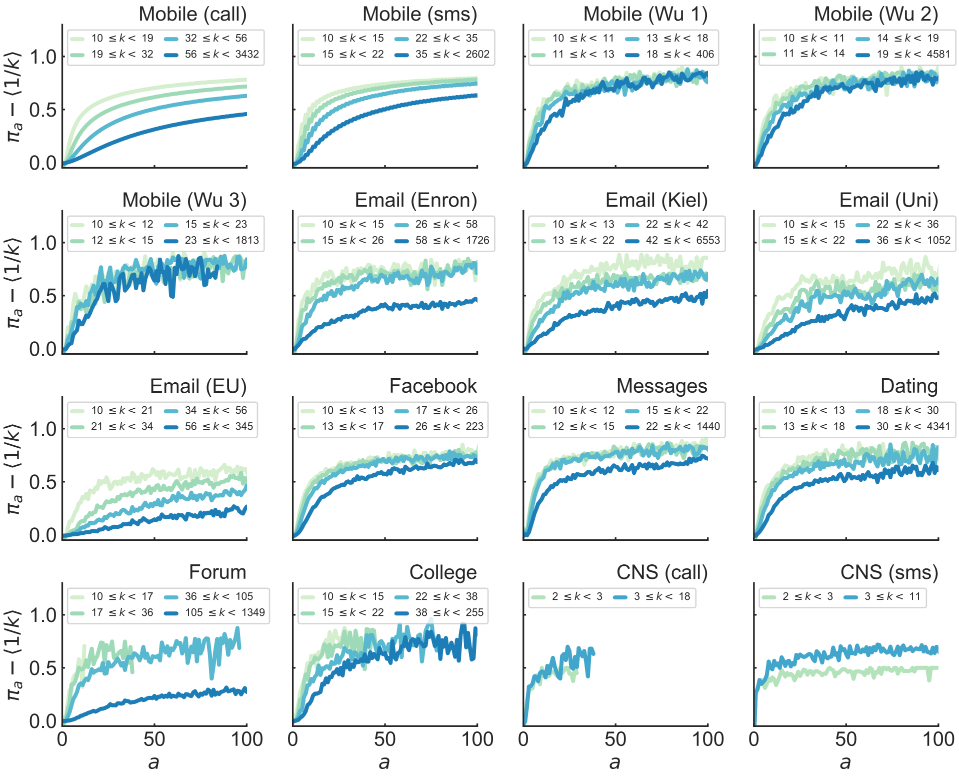


**Figure S5. Connection kernel by degree.** Relative connection kernel $\pi_a - \langle 1/k \rangle$ as a function of alter activity $a$ in all datasets, averaged over time for egos with degree $k$ in interval [each $a$ value corresponds to at least 10 egos with $k \geq 10$, or $k \geq 2$ for CNS (call, sms)]. The baseline at 0 denotes the case where communication events are distributed randomly. Lines correspond to the quartile ranges of the degree distribution (see Fig. S1), or to 2-quantile ranges for CNS (call, sms). In all degree intervals, the growth of the connection kernel $\pi_a$ with activity $a$ indicates that alters of high prior activity receive more communication. Smaller ego networks (low degree $k$) show slightly higher levels of cumulative advantage.

In order to measure the variability in communication patterns between egos and alters (the heterogeneity of tie strengths in an ego network), we focus on the alter activity distribution $p_a$, the probability that a randomly chosen alter has activity $a$. Following [26], we quantify the spread of $p_a$ via the variance-to-mean ratio $\sigma_r^2/\mu_r$ by defining the dispersion index

$$d = \frac{\sigma_r^2/\mu_r - 1}{\sigma_r^2/\mu_r + 1} = \frac{\sigma_r^2 - \mu_r}{\sigma_r^2 + \mu_r} \tag{S1}$$

for each ego network in a dataset, where $\mu_r = t_r = t - a_0$ is the mean alter activity relative to the minimum $a_0$, and $\sigma_r^2 = \sigma^2$ is the (location-invariant) variance of alter activity. The CCDF $P[d' \geq d]$ (fraction of egos having at least dispersion index $d$) varies smoothly with $d$ in all systems, meaning there are egos with both narrow ($d \sim 0$) and broad ($d \sim 1$) alter activity distributions (Fig. S2). This variability in how individuals distribute communication events is also apparent by calculating the CCDF $P[a' \geq a]$ of the number of alters having at least activity $a$, restricted to ego networks in each quartile range of the dispersion distribution $p_d$ (Fig. S3). Egos with larger $d$ have both a broader activity distribution $p_a$, following Eq. (S1), and higher alter activities overall.

We also calculate the connection kernel $\pi_a$, the average probability that an alter with current activity $a$ communicates once more with the ego. After averaging over time and large enough subsets of ego-alter pairs with activity $a$ at some point in time, the connection kernel $\pi_a$ increases with alter activity

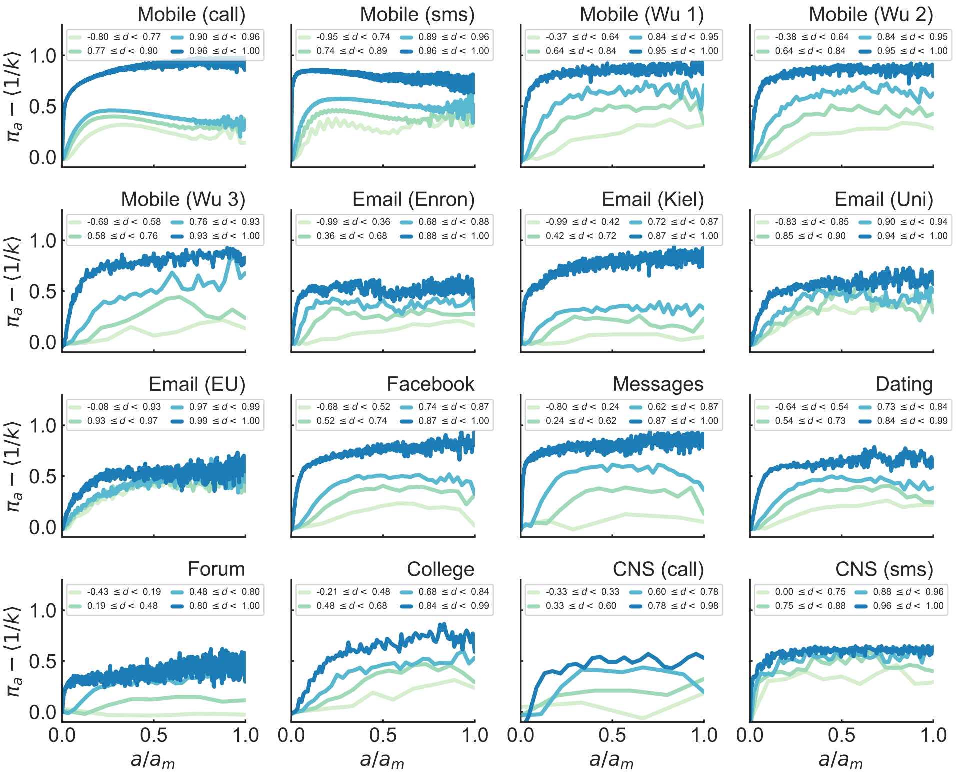


**Figure S6. Connection kernel by dispersion.** Relative connection kernel $\pi_a - \langle 1/k \rangle$ as a function of normalized alter activity $a/a_m$ in all datasets, averaged over time for egos with dispersion $d$ in interval, where $a_m$ is the maximum alter activity of the selected egos [each $a$ value corresponds to at least 30 egos with $k \geq 10$, or $k \geq 2$ for CNS (call, sms)]. The baseline at 0 denotes the case where communication events are distributed randomly. Lines correspond to the quartile ranges of the dispersion distribution (see Fig. S2). Dispersion is strongly associated with the level of cumulative advantage in the ego network. For large dispersion, the growth of the connection kernel $\pi_a$ with activity $a$ indicates that alters of high prior activity receive more communication. For low dispersion, communication events happen more at random.

(Fig. S4). Apart from low values of $a$, $\pi_a$ is larger than the average baseline $\langle 1/k \rangle$ (communication events are distributed uniformly at random among alters) and increases roughly monotonically for all datasets, with a concave shape where the slope of change depends on the activity range (i.e. the slope is high for low activity, and tends to decrease for larger values of $a$). This behavior indicates cumulative advantage: alters with high prior activity are more likely to communicate with the ego later on in time.

Cumulative advantage, as signaled by a growing connection kernel, is evident not only on the system as a whole, but also when controlling for some properties of the ego network, namely degree and dispersion. We compute the relative connection kernel $\pi_a - \langle 1/k \rangle$ for subsets of egos in each quartile range of both degree and dispersion distributions (Figs. S5-S6; see Figs. S1-S2 for the underlying distributions). Regardless of degree, all ego networks show signals of cumulative advantage, with the same concave functional form for $\pi_a$ as in the entire system (compare Fig. S5 with Fig. S4). For most datasets, the level of cumulative advantage is slightly higher in smaller ego networks. In turn, dispersion is strongly associated with the level of cumulative advantage in the ego network (Fig. S6). When tie strengths are heterogeneous and the alter activity distribution $p_a$ is broad (large $d$), we see a similar growing connection kernel as for all degree groups and the whole system. But as $d$ goes down and alters become more homogeneous in their activity levels, $\pi_a$ approaches the baseline $\langle 1/k \rangle$, implying that communication events are distributed at random among alters. For low dispersion, the connection kernel first increases

with activity and then remains roughly constant or even decreases. These results suggest a relationship between an underlying mechanism for cumulative advantage and the emergence of heterogeneous alter activity, which we explore in what follows via modeling.

# S2 Model of alter activity

Consider a social ego network made up of one central individual (the ego) and its $k$ acquaintances (the alters), where a tie between ego and alter represents communication activity between individuals (i.e. calls/messages or online interactions). At a discrete event time $\tau$ (starting from $\tau_0$ up to the length of the observation window), each alter $i = 1, \ldots, k$ has an activity score $a_i(\tau)$ counting the number of times the ego and alter $i$ have communicated until time $\tau$. We take as initial condition $a_i(\tau_0) = a_0$ for all $i$, meaning that all alters have the same initial activity $a_0 \geq 0$, i.e. the minimum activity observed across alters. At each time $\tau$ of the model dynamics, a single alter $i$ communicates with the ego, such that $a_i(\tau + 1) = a_i(\tau) + 1$. Taking $\tau_0 = ka_0$, we ensure that event time is equal to the sum of all communication events in the ego network, i.e. $\tau = \sum_i a_i$ is the total communication activity. Scores are thus bounded by the growing interval $a_i(\tau) \in [\tau_0, \tau]$.

We consider a cumulative-advantage dynamics (similar to Price's model [27–29]) tuned by a parameter $\alpha$: the probability $\pi_a(\tau)$ that an alter with previous activity $a_i(\tau) = a$ is active at time $\tau+1$ is proportional to its past number of communications,

$$\pi_a(\tau) = \frac{a + \alpha}{\sum_j [a_j(\tau) + \alpha]} = \frac{a + \alpha}{\tau + k\alpha}. \tag{S2}$$

The connection kernel in Eq. (S2) is well defined (at any time $\tau \geq \tau_0$) for any $\alpha$ larger than its minimum value $\alpha_0 = -a_0$, so we can also tune the model by the relative parameter $\alpha_r = \alpha - \alpha_0 = \alpha + a_0 > 0$. Similarly, we define the relative alter activity $a_r = a - a_0$, relative event time $\tau_r = \tau - \tau_0 = \tau - ka_0$, and the relative mean alter activity $t_r = t - t_0 = t - a_0$ with $t = \tau/k$. Introducing the preferentiality parameter

$$\beta = \frac{t_r}{\alpha_r} = \frac{t - a_0}{\alpha + a_0} \tag{S3}$$

allow us to rewrite Eq. (S2) as

$$\pi_a = \frac{a_r/t_r + \beta^{-1}}{k(1 + \beta^{-1})}. \tag{S4}$$

As we will see in Section S2.1, the scale $\beta$ (or, alternatively, the rate $\beta^{-1}$) quantifies a crossover between regimes of behavior in alter activity. For $\beta \ll 1$ (i.e. $\alpha \to \infty$ for fixed $t$ and $a_0$), we have $\pi_a = 1/k$ for any $a_r$ and communication events are spread uniformly at random among alters. For $\beta \gg 1$ (i.e. $\alpha \to \alpha_0$ for fixed $t$ and $a_0$), the probability of communication is roughly proportional to activity, $\pi_a \to a_r/\tau_r$. In this way, the preferentiality parameter $\beta$ interpolates between a *homogeneous regime* where communication in the ego network is uniformly random ($\beta < 1$), and a *heterogeneous regime* where activity is driven by cumulative advantage ($\beta > 1$), with a crossover at $\beta = 1$ ($\alpha_r = t_r$).

## S2.1 Master equation for activity dynamics

We treat our model analytically by solving a master equation for the activity dynamics in the limit of large total alter activity $\tau \to \infty$ and large number of alters $k \to \infty$, such that the mean alter activity $t = \tau/k$ is kept constant. We denote by $p_a(\tau)$ the time-dependent probability that an alter chosen uniformly at random has activity $a$ at time $\tau$, i.e. the alter activity distribution. When a new communication event happens at time $\tau + 1$, with probability $\pi_a$ the group of $kp_a$ alters with activity $a$ loses one alter (since the alter's activity increases to $a + 1$). With probability $\pi_{a-1}$ the group also wins one alter from the

group of $kp_{a-1}$ alters with activity $a-1$ (since the alter's activity increases to $a$). The master equation for $p_a$ is

$$p_a(\tau+1) - p_a(\tau) = \pi_{a-1}(\tau) p_{a-1}(\tau) - \pi_a(\tau) p_a(\tau), \tag{S5}$$

with initial condition $p_a(\tau_0) = \delta_{a,a_0}$, and $p_a \equiv 0$ for $a < a_0$.

Taking the limit $\tau, k \to \infty$ (with $dt = 1/k \to 0$) and rescaling time to the fixed mean alter activity $t = \tau dt$, we can rewrite Eq. (S5) as a continuous master equation for the alter activity distribution $p_a(t)$,

$$d_t p_a = \frac{1}{t+\alpha} \big[ (a-1+\alpha) p_{a-1} - (a+\alpha) p_a \big], \tag{S6}$$

with $d_t$ the derivative with respect to $t$. The initial time is $t_0 = \tau_0/k = a_0$, so the initial condition of Eq. (S6) is $p_a(t_0) = \delta_{a,a_0}$, with $p_a \equiv 0$ for $a < a_0$. We solve Eq. (S6) within a generating function formalism. We introduce the probability generating function (PGF) $g(z,t)$ associated to $p_a$,

$$g(z,t) = \sum_{a=0}^{\infty} p_a(t) z^a. \tag{S7}$$

which returns the probability $p_a$ by computing the $a$-th partial derivative with respect to $z$, $p_a(t) = \partial_z^a g(0,t)/a!$. Summing up over $a$ in Eq. (S6) and manipulating dummy indices, we obtain a partial differential equation (PDE) for $g$,

$$\partial_t g = \frac{z-1}{t+\alpha} \big( z \partial_z g + \alpha g \big), \tag{S8}$$

with initial condition $g(z, t_0) = z^{a_0}$.

The linear PDE in Eq. (S8) can be solved with the method of characteristics. By introducing an auxiliary variable $s$, solving Eq. (S8) is equivalent to solving the system of ordinary differential (Lagrange-Charpit) equations for $t \equiv t(s)$, $z \equiv z(s)$ and $g \equiv g(s)$,

$$\begin{cases} d_s t = t + \alpha, & t(0) = a_0, \\ d_s z = z(1-z), & z(0) = z_0, \\ d_s g = \alpha(z-1) g, & g(0) = z_0^{a_0}. \end{cases} \tag{S9}$$

Using the solutions of Eq. (S9) to substitute $s$ and $z_0$, we obtain an explicit expression for the PGF,

$$g(z,t) = z^{a_0} \left[ z + (1-z) \frac{t+\alpha}{a_0+\alpha} \right]^{-(a_0+\alpha)} = z^{a_0} \left[ z + (1-z)(1+\beta) \right]^{-\alpha_r}, \tag{S10}$$

where we use the preferentiality parameter $\beta = t_r/\alpha_r$ (with $\alpha_r = \alpha + a_0$ and $t_r = t - a_0$).

Equating terms between Eq. (S7) and the Mclaurin series of Eq. (S10) lets us calculate the alter activity distribution $p_a$ explicitly by calculating partial derivatives of the PGF $g$ with respect to $z$. After some algebra and by using the preferentiality parameter $\beta$ we obtain

$$p_a(t) = p_0 \frac{a_r^{-1}}{\mathrm{B}(a_r, \alpha_r)} \left( 1 + \frac{1}{\beta} \right)^{-a_r} \tag{S11}$$

for $a_r > 0$ ($a > a_0$), with

$$p_0 = p_{a_0}(t) = (1+\beta)^{-\alpha_r} \tag{S12}$$

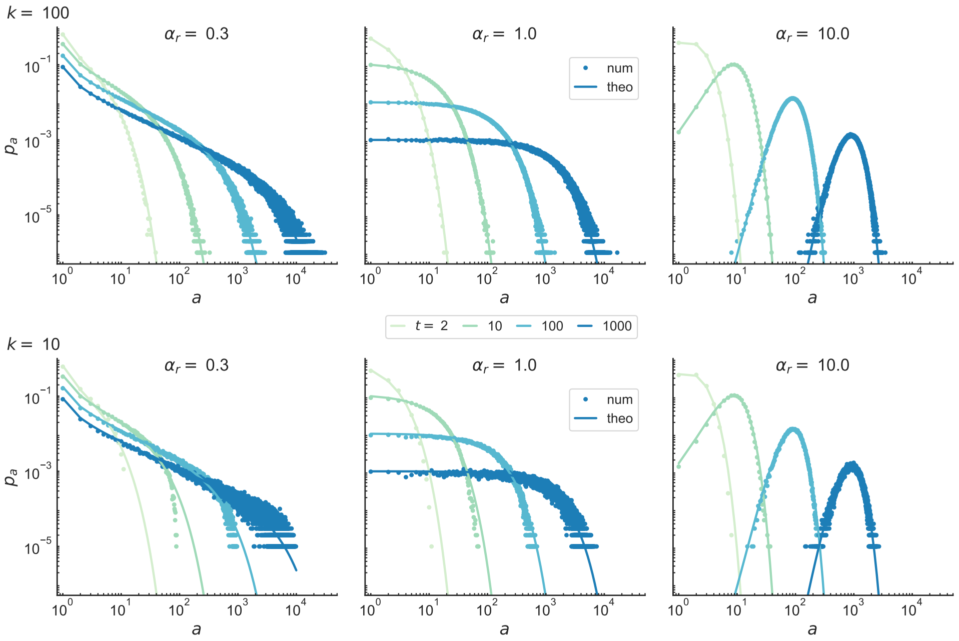


**Figure S7. Simple model of alter activity.** Probability $p_a(t)$ of a randomly selected alter having activity $a$ at time $t$, as a function of $a = a_r + a_0$ for varying $t = t_r + a_0$ and varying $\alpha_r = \alpha + a_0$, for fixed $a_0 = 1$ and $k = 10, 100$ (bottom/top rows), in both numerical simulations of the model [Eq. (S2); dots] and its analytical solution [Eq. (S11); lines]. For given $t$ and $a_0$, the time evolution of $p_a$ [as defined by Eq. (S6)] reaches an $\alpha_r$-dependent asymptotic shape. **(right)** When $\alpha_r \to \infty$ ($\alpha \to \infty$), $p_a$ converges to a Poisson distribution with mean and variance $t_r$ [Eq. (S16)]. **(left)** When $\alpha_r \to 0$ ($\alpha \to -a_0$), $p_a$ approaches a gamma distribution with shape $\alpha_r$ and scale $\beta = t_r/\alpha_r$ [Eq. (S14)]. **(middle)** When $\alpha_r = 1$, the exponent of the power-law decay in the gamma distribution is $\alpha_r - 1 = 0$, so the activity distribution has a plateau of $a$ values with relatively constant $p_a$ that grows with $t$. Eq. (S11) approximates numerical simulations very well, but fails at the tail for sufficiently low $k$. Simulations are averaged over $10^4$ realizations.

for $a_r = 0$ ($a = a_0$) and $p_a \equiv 0$ for $a_r < 0$ ($a < a_0$). For consistency, $p_a(t) = \delta_{a,a_0}$ for $t_r = 0$ ($t = a_0$). In Eq. (S11), $\mathrm{B}(a_r, \alpha_r) = \Gamma(a_r)\Gamma(\alpha_r)/\Gamma(a_r + \alpha_r)$ is the Euler beta function, with $\Gamma(a_r) = (a_r - 1)!$ the gamma function.

The $n$-th raw moment of $p_a$ can also be computed from Eq. (S10) as $m^{(n)} = (z\partial_z)^n g|_{z=1}$, leading to the mean $\mu = t$ (in consistence with the definition of the model) and variance $\sigma^2 = t_r(1 + \beta)$. Changing variables from $a$ to the relative alter activity $a_r = a - a_0$, we obtain the relative mean $\mu_r = t_r$ and variance $\sigma_r^2 = \sigma^2$ (since variance is location-invariant). This allows us to write the dispersion index $d$ of Eq. (S1) in terms of $\beta$ as

$$d = \frac{\sigma_r^2 - \mu_r}{\sigma_r^2 + \mu_r} = \frac{\beta}{2 + \beta}. \tag{S13}$$

Eq. (S11) has an intuitive behaviour as a function of the relative alter activity $a_r$, mean alter activity $t_r$, cumulative-advantage parameter $\alpha_r$, and the minimum alter activity $a_0$ (Fig. S7). Even if the derivation of Eq. (S11) assumes $\tau, k \to \infty$ for fixed $t$, its functional form agrees very well with numerical simulations of the dynamical rule in Eq. (S2) for degree as low as $k = 100$ (Fig. S7 upper row), with some disagreement in the tail of the activity distribution for even lower $k = 10$ due to finite-size effects (Fig. S7 lower row). The first factor in Eq. (S11), $p_0$, shows that the fraction of alters with minimum activity decreases as time goes by with a decay regulated by $\alpha_r$. The second factor, $a_r^{-1}/\mathrm{B}(a_r, \alpha_r)$, is roughly a power law for intermediate values of activity $a_r$ with exponent regulated by $\alpha_r$. The third

factor is an exponential cutoff for large activity $a_r$ at the scale $\beta$ that moves to the right as time $t_r$ increases. As we will see below, the behaviour of the activity distribution $p_a(t)$ is even more apparent by approximating Eq. (S11) in the heterogeneous ($\beta > 1$) and homogeneous ($\beta < 1$) regimes by either a gamma or Poisson distribution. Since these distributions have their own scaling form, $\beta$ parametrises a crossover between regimes in terms of the scaling of the activity distribution.

#### S2.1.1 Heterogeneous regime ($\beta > 1$): Alter activity is gamma-distributed

We explore the limit $\alpha_r \to 0$ ($\alpha \to -a_0$ for fixed $a_0$) by considering a large activity $a_r \gg 0$ ($a \gg a_0$, i.e. the tail of the activity distribution) for small but fixed $\alpha_r$. Then, the beta function behaves as $\mathrm{B}(a_r, \alpha_r) \simeq \Gamma(\alpha_r) a_r^{-\alpha_r}$ for given $\alpha_r$. The condition $\beta > 1$ leads to the approximations $(1+\beta)^{-\alpha_r} \simeq \beta^{-\alpha_r}$ and $(1+1/\beta)^{-a_r} \simeq e^{-a_r/\beta}$ (from a 1st-order Taylor expansion of the exponential). Inserting into Eq. (S11) we obtain

$$p_a(t) = \frac{1}{\beta^{\alpha_r}\Gamma(\alpha_r)} a_r^{\alpha_r - 1} e^{-a_r/\beta}, \quad \alpha_r \to 0, \tag{S14}$$

a gamma distribution with shape $\alpha_r$ and scale $\beta$. Then, the relative alter activity $a_r$ has mean $t_r$ and variance $t_r\beta$. Consistently, $\sigma_r^2 \to \infty$ as $\beta \to \infty$, implying a dispersion $d \to 1$ [see Eq. (S13)]. In the heterogeneous regime where alters communicate with the ego with probability proportional to their previous activity, the activity distribution $p_a(t)$ has power-law behaviour with exponent $\alpha_r - 1$ and an exponential cutoff regulated by the scale $\beta$ (see, e.g., Fig. S7 left).

The moment-generating function of the gamma distribution shows that Eq. (S14) has exponential scaling. Plugging the rescaled activity $a'_r = a_r/\beta$ into Eq. (S14) leads to

$$\beta p_a(t) = \frac{1}{\Gamma(\alpha_r)} \left(\frac{a_r}{\beta}\right)^{\alpha_r - 1} e^{-a_r/\beta}, \tag{S15}$$

the standard gamma distribution (with shape $\alpha_r$ and scale 1). In a plot of $\beta p_a$ vs. $a_r/\beta$ for varying $t_r$ and fixed $\alpha_r$, all curves collapse to the standard form of Eq. (S15) (Fig. S8 top row). The gamma distribution (and its scaling property) is a very good approximation of the activity distribution even for relatively low activity $a_r$, as long as we are in the heterogeneous regime of $\beta > 1$ (for example, gamma scaling fails in the top right plot of Fig. S8 for $t = 2$ and $\alpha_r = 10$ since $\beta = 0.1$). The gamma scaling shape in the heterogeneous regime depends on $\alpha_r$, but remains a good approximation of the activity distribution even at the crossover $\beta = 1$.

#### S2.1.2 Homogeneous regime ($\beta < 1$): Alter activity is Poisson-distributed

In the limit $\alpha_r \to \infty$ ($\alpha \to \infty$ for fixed $a_0$) where alters communicate with the ego uniformly at random, the beta function behaves as $\mathrm{B}(a_r, \alpha_r) \simeq \Gamma(a_r)\alpha_r^{-a_r}$ for given $a_r$. For $\beta < 1$, we can approximate $(1+\beta)^{-\alpha_r} \simeq e^{-t_r}$ (from a 1st-order Taylor expansion of the exponential) and $(1+1/\beta)^{-a_r} \simeq \beta^{a_r}$. Then, the activity distribution converges to a Poisson distribution with mean and variance $t_r$,

$$p_a(t) = \frac{t_r^{a_r} e^{-t_r}}{a_r!}, \quad \alpha_r \to \infty. \tag{S16}$$

Thus, for large $\alpha_r$, the decay of the activity distribution $p_a$ is exponential and independent of $\alpha_r$ (Fig. S7 right panel). Since $\sigma_r^2 \to t_r$ as $\beta \to 0$, this limit consistently recovers a dispersion $d \to 0$ [Eq. (S13)].

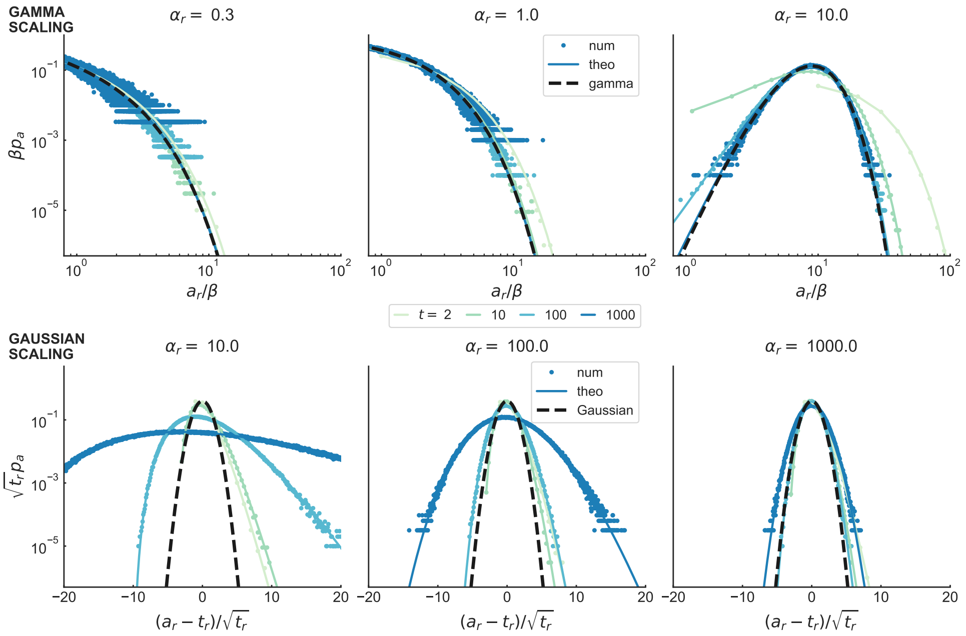


**Figure S8. Crossover in scaling of alter activity.** Probability $p_a(t)$ of a randomly selected alter having activity $a$ at time $t$, as a function of $a_r = a - a_0$ for varying $t = t_r + a_0$ and varying $\alpha_r = \alpha + a_0$, for fixed $a_0 = 1$ and $k = 100$, in both numerical simulations of the model [Eq. (S2); dots] and its analytical solution [Eq. (S11); lines]. **(top)** By plotting $\beta p_a$ vs. $a_r/\beta$ for varying $t_r$ and fixed $\alpha_r$, curves collapse to the standard gamma distribution [Eq. (S15); dashed lines]. This $\alpha_r$-dependent, gamma scaling is valid in the heterogeneous regime $\beta > 1$, with $\beta = t_r/\alpha_r$ the scale parameter of the gamma distribution in Eq. (S14). Though only asymptotically correct, gamma scaling is a good approximation even at the crossover $\beta = 1$. **(bottom)** By plotting $\sqrt{t_r} p_a$ vs. $(a_r - t_r)/\sqrt{t_r}$ for varying $t_r$, curves collapse to the standard Gaussian distribution [Eq. (S17); dashed lines]. This Gaussian scaling is valid in the homogeneous regime $\beta < 1$ and becomes asymptotically more accurate with $t_r$. Simulations are averaged over $10^4$ realizations.

The scaling behaviour of the Poissonian activity distribution in Eq. (S16) is apparent from exploring the limit $t_r \to \infty$ (i.e. large mean alter activity $t$ for fixed $a_0$). Using Stirling's approximation of the gamma function, $a_r! = \Gamma(a_r + 1) \simeq \sqrt{2\pi a_r} e^{-a_r} a_r^{a_r}$, and assuming that $p_a$ only takes significant values close to $a_r = t_r$, the activity distribution approaches a Gaussian distribution with mean $t_r$ and standard deviation $\sqrt{t_r}$,

$$p_a(t) = \frac{1}{\sqrt{2\pi t_r}} e^{-\frac{(a_r - t_r)^2}{2t_r}}, \quad t_r \to \infty. \tag{S17}$$

When plotting $\sqrt{t_r} p_a$ vs. $(a_r - t_r)/\sqrt{t_r}$ for varying $t_r$, all curves collapse to the standard Gaussian distribution with mean 0 and standard deviation 1 (Fig. S8 bottom row). The Poisson distribution (and its Gaussian scaling property) is a very good approximation of the activity distribution even for relatively low $t_r$, as long as we are in the homogeneous regime of $\beta < 1$ (for example, Gaussian scaling fails in the bottom center plot of Fig. S8 for $t = 1000$ and $\alpha_r = 100$ since $\beta = 10$). Note that the asymptotic Gaussian scaling shape in the homogeneous regime is independent of $\alpha_r$, but it converges slowly as we increase $t_r$.

Overall, the model of alter activity in social ego networks defined by the dynamical rule in Eq. (S2) has two regimes of behavior in $(\alpha_r, t_r)$-space regulated by the preferentiality parameter $\beta = t_r/\alpha_r$ (Fig. S9 top). In the homogeneous regime of $\beta < 1$, the activity distribution $p_a(t)$ is asymptotically Poissonian with Gaussian scaling for increasing $\alpha_r$ and $t_r$, meaning that the ego spreads events homogeneously across its alters, with no strong dependence on the particular value of $\alpha_r$. In the heterogeneous regime

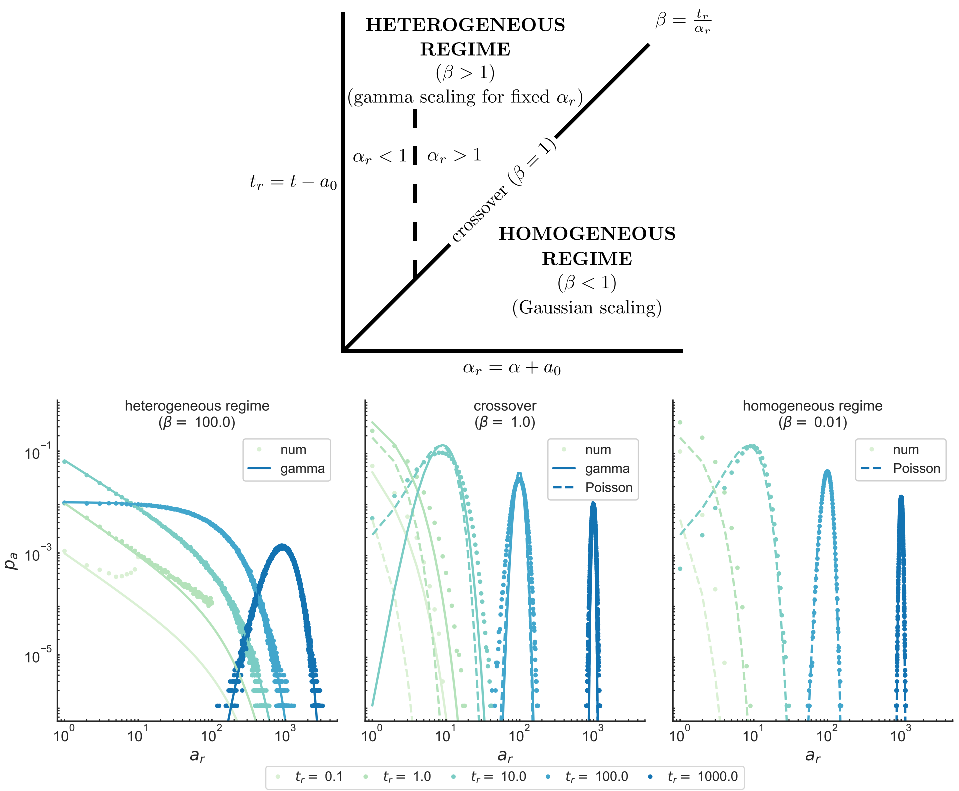


**Figure S9. Scaling regimes in alter activity. (top)** Phase diagram in $(\alpha_r, t_r)$-space, showcasing the scaling regimes in alter activity of the model defined by Eq. (S2). **(bottom)** Probability $p_a(t)$ of a randomly selected alter having activity $a$ at time $t$, as a function of $a_r = a - a_0$ for varying $t_r = t - a_0$ and varying $\alpha_r = \alpha + a_0$ such that $\beta = t_r/\alpha_r$ is constant, for fixed $a_0 = 1$ and $k = 100$, in both numerical simulations of the model [Eq. (S2); dots] and the gamma [Eq. (S14)] and Poisson [Eq. (S16)] approximations. When $\beta < 1$ (right), activity is homogeneously distributed across alters and $p_a(t)$ is Poissonian with asymptotic Gaussian scaling. When $\beta > 1$ (left), $p_a(t)$ has gamma scaling for varying $t_r$ and fixed $\alpha_r$. For $\alpha_r < 1$ a few alters accumulate most activity, and for $\alpha_r > 1$ alter activity is more homogeneously distributed. Regimes are separated by a crossover at $\beta = 1$ where both gamma and Gaussian scaling forms fail slightly.

of $\beta > 1$, $p_a(t)$ is well approximated by a gamma distribution, meaning that the activity distributions for varying $t_r$ and fixed $\alpha_r$ scale together (vertical lines in the upper triangle of Fig. S9 top). The activity distribution is either monotonically decreasing for $\alpha_r < 1$ (a few alters accumulate most events) or has a broad peak around $t_r$ for $\alpha_r > 1$ (alter activity is a bit more homogeneous). When $\beta = 1$, both gamma and Gaussian scaling forms fail to reproduce numerical simulations slightly (Fig. S9 bottom), implying a crossover between the heterogeneous and homogeneous regimes. The largest disagreement with simulations occurs for really low $t_r$ and $\alpha_r$, due to the assumption $\tau, k \to \infty$ in the derivation of Section S2.1.

# S3 Fitting data and model

## S3.1 Derivation of maximum likelihood estimates

The analytical derivation of the activity distribution $p_a(t)$ in the limit $\tau, k \to \infty$ for fixed mean alter activity $t$ and given minimum alter activity $a_0$ (see Section S2.1) allows us to write explicitly a maximum likelihood estimate (MLE) for the model parameter $\alpha$ associated with an empirical observation of alter activities. Take a finite ego network of $k$ alters observed during a window of length $t_r = t - a_0$, where each alter $i$ has $a_i$ communication events with the ego ($i = 1, \dots, k$), and the least active alter has $a_0$ events [1]. Since in empirical data we recognize alters when there is at least a single communication event with the ego, we always measure $a_0 > 0$. The total alter activity is $\tau = \sum_i a_i$ and the empirical mean alter activity is $t = \tau/k$, so the only free parameter in the model is $\alpha$. Assuming that alter activities are independent and identically distributed random variables following the activity distribution $p_a$ in Eqs. (S11)-(S12), the likelihood $L_\alpha$ that the sample $\{a_i\}$ is generated by $p_a$ for a certain $\alpha$ value is given by the product

$$L_\alpha = \prod_{i=1}^{k} p_{a_i}(t) = p_0^k \prod_{a_r \neq 0} \frac{a_r^{-1}}{\mathrm{B}(a_r, \alpha_r)} \left(1 + \frac{1}{\beta}\right)^{-a_r}, \tag{S18}$$

with $p_0 = (1+\beta)^{-\alpha_r}$ from Eq. (S12), where we use the relative quantities $a_r = a_i - a_0$, $\alpha_r = \alpha + a_0$, and the preferentiality parameter $\beta = t_r/\alpha_r$. Like in Eq. (S2), we have the constraints $\alpha_r > 0$ and $a_r \geq 0$ for all $i$.

Taking the natural logarithm of Eq. (S18) and its derivative with respect to $\alpha$ leads, after some algebra, to

$$d_\alpha \ln L_\alpha = k \left[ F_\alpha - \ln(1+\beta) \right] \tag{S19}$$

where $F_\alpha = \frac{1}{k} \sum_i [\psi(a_r + \alpha_r) - \psi(\alpha_r)]$ is an average over all observed relative activities $a_r = a_i - a_0$ of the digamma function $\psi(\alpha) = d_\alpha \Gamma(\alpha)/\Gamma(\alpha)$, i.e. the logarithmic derivative of the gamma function $\Gamma(\alpha)$. The MLE $\hat{\alpha}$ that maximizes $L_\alpha$ with respect to $\alpha$ (or, alternatively, the MLE $\hat{\beta} = t_r/\hat{\alpha}_r$ with $\hat{\alpha}_r = \hat{\alpha} + a_0$), is given implicitly by

$$d_\alpha \ln L_\alpha |_{\alpha = \hat{\alpha}} = 0. \tag{S20}$$

Eqs. (S19)-(S20) lead to a trascendental equation for the estimated cumulative-advantage parameter $\hat{\alpha}$,

$$\hat{\alpha}_r = \frac{t_r}{e^{F_{\hat{\alpha}}} - 1}, \tag{S21}$$

or, equivalently, $\hat{\beta} = e^{F_{\hat{\alpha}}} - 1$ for the estimated preferentiality parameter $\hat{\beta}$. Observe that Eq. (S21) is valid only if $a_i > a_0$ for at least some alter $i$, so that the empirical activity distribution is different from the initial condition in the model $[p_a(t_0) = \delta_{a,a_0}]$. When all alters have the same activity [i.e. $a_i = a_0$ for all $i$, so $a_r = t_r = \beta = F_\alpha = 0$ for nonzero $\alpha_r$], Eq. (S20) is trivially valid for any $\alpha$ and we cannot use the MLE procedure to find an optimal value. This is consistent with the filtering condition $t > a_0$ for empirical communication data introduced in Section S1, implying that we can potentially find MLEs for all considered ego networks.

We can verify the accuracy of the MLE method by solving Eq. (S21) graphically and numerically for synthetic activity distributions $p_a(t)$ coming from many stochastic realizations of the model rule in

[1]Note that the results of Section S2.1 are strictly valid only in the limit $\tau, k \to \infty$, but we use them to obtain MLEs in empirical ego networks with finite (and heterogeneously distributed) degree $k$.

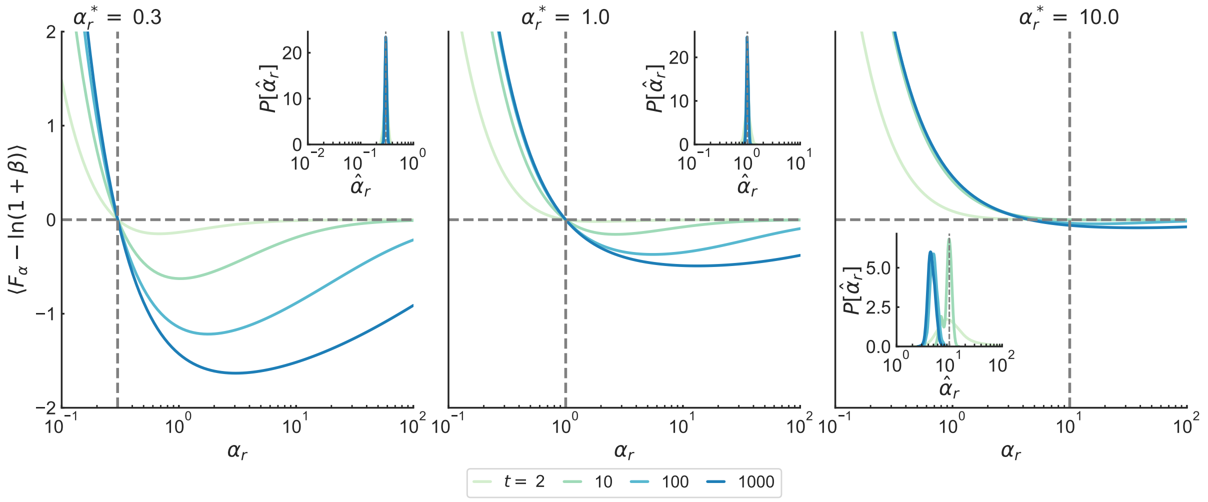


**Figure S10. Consistency of maximum likelihood estimation.** Numerical simulations of alter activity [according to Eq. (S2)] for several target values $\alpha_r^*$ of the model parameter and varying $t = t_r + a_0$, with $a_0 = 1$ and $k = 10^3$. The quantity $\langle F_\alpha - \ln(1+\beta)\rangle$ as a function of $\alpha_r$ (an average over $10^3$ simulations), has a root $\hat{\alpha}_r$ somewhere close to $\alpha_r^*$, in accordance with Eq. (S21). Insets show the kernel density estimate $P[\hat{\alpha}_r]$ of the MLE $\hat{\alpha}_r$ [as computed numerically from Eq. (S21)] over all simulations, which is centered around $\alpha_r^*$ for most parameter values. The MLE procedure recovers the target value $\alpha_r^*$ and is thus consistent, apart from a systematic underestimation for large $t$ in the homogeneous regime.

Eq. (S2) (Fig. S10). We obtain samples $\{a_i\}$ from numerical simulations of the model for given $k$, $t$, and $a_0$, for several target values $\alpha_r^*$ of the model parameter. Then we plot the average $\langle F_\alpha - \ln(1+\beta)\rangle$ over all realizations as a function of $\alpha_r$ to graphically locate a root, and we also compute $\hat{\alpha}_r$ numerically from Eq. (S21), which follows the distribution $P[\hat{\alpha}_r]$ over all realizations (inset in Fig. S10). The MLE procedure is quite accurate ($\hat{\alpha}_r \sim \alpha_r^*$) and thus consistent in the heterogeneous and crossover regimes. In the homogeneous regime, however, $\hat{\alpha}_r$ systematically underestimates the target value $\alpha_r^*$ for large $t$, where the functional form of $\langle F_\alpha - \ln(1+\beta)\rangle$ does not depend much on $t$ anymore. It is instructive to see this MLE bias in light of the scaling property of the activity distribution. In the homogeneous regime, alter activities asymptotically scale like a Gaussian regardless of the value of $\alpha$, so even relatively large errors in estimating $\alpha$ lead to the same scaling form. In the heterogeneous regime, where activities have an $\alpha$-dependent gamma scaling, less bias means we can estimate the scaling form of empirical data more accurately.

## S3.2 Goodness-of-fit test

The MLE $\hat{\alpha}$ given implicitly by Eq. (S21) is the value of $\alpha$ maximizing the likelihood that the activity model of Section S2 produces a given empirical activity distribution. In addition, we need a goodness-of-fit (GOF) test quantifying how plausible is the hypothesis that the empirical data is drawn from the theoretical activity distribution $p_a(t)$ in Eq. (S11). Following [30], we measure goodness of fit by means of the distance between the activity distributions in model and data. (We have previously used this method to gauge the plausibility of several models of rank distributions in sports performance data [31]; for a rigorous criticism of the methods of [30] based on extreme value theory, see [32].) We choose as distance metric four different test statistics [33]. The first one is the standard Kolmogorov-Smirnov (KS) statistic [34],

$$D = \max_{a_0 \le a \le a_m} |\Delta P_a|, \tag{S22}$$

| Dataset | $N$ | $n_D$ | $n_{W^2}$ | $n_{U^2}$ | $n_{A^2}$ |
|---|---|---|---|---|---|
| Mobile (call) | 5431921 | 0.70 | 0.69 | 0.65 | 0.69 |
| Mobile (sms) | 4233187 | 0.63 | 0.69 | 0.69 | 0.63 |
| Mobile (Wu 1) | 16050 | 0.42 | 0.61 | 0.70 | 0.43 |
| Mobile (Wu 2) | 20534 | 0.38 | 0.58 | 0.68 | 0.38 |
| Mobile (Wu 3) | 4215 | 0.36 | 0.52 | 0.62 | 0.37 |
| Email (Enron) | 21984 | 0.39 | 0.49 | 0.55 | 0.40 |
| Email (Kiel) | 9842 | 0.33 | 0.42 | 0.49 | 0.34 |
| Email (Uni) | 2456 | 0.71 | 0.78 | 0.75 | 0.70 |
| Email (EU) | 866 | 0.62 | 0.58 | 0.54 | 0.60 |
| Facebook | 31429 | 0.59 | 0.65 | 0.67 | 0.59 |
| Messages | 20252 | 0.39 | 0.43 | 0.45 | 0.38 |
| Dating | 16239 | 0.56 | 0.65 | 0.67 | 0.57 |
| Forum | 4122 | 0.43 | 0.46 | 0.46 | 0.42 |
| College | 1303 | 0.65 | 0.67 | 0.66 | 0.64 |
| CNS (call) | 285 | 0.34 | 0.52 | 0.68 | 0.34 |
| CNS (sms) | 347 | 0.54 | 0.81 | 0.88 | 0.54 |

**Table S2. Statistical significance of maximum likelihood estimation**. Fraction $n_\bullet$ of ego networks satisfying the condition $p_\bullet > 0.1$ on the $p$-value $p_\bullet$ associated to the test statistics of Kolmogorov-Smirnov, Cramér-von Mises, Watson, and Anderson-Darling [$\bullet = D, W^2, U^2, A^2$, respectively; see Eqs. (S22)-(S25)]. Fractions $n_\bullet$ are calculated relative to the number $N$ of egos in each dataset under the condition $t > a_0$ (i.e. with any level of heterogeneity on their communication signatures). The model is able to reproduce observed data for most egos, at least according to some statistic. For large datasets, statistical significance is robust to the choice of statistic.

that is, the largest magnitude of the difference $\Delta P_a(t) = P_{\text{data}}[a' \leq a] - P_a(t)$ between the cumulative distribution function (CDF) in data, $P_{\text{data}}[a' \leq a]$, and the CDF of the fitted model, $P_a(t) = \sum_{a'=a_0}^{a} p_{a'}(t)$, across all activities $a \in [a_0, a_m]$, where $a_0$ and $a_m$ are the minimum and maximum alter activities in the empirical ego network, respectively. The other three belong to the Cramér-von Mises family of test statistics [35–38]: the Cramér-von Mises ($W^2$) statistic,

$$W^2 = k \sum_{a=a_0}^{a_m} \Delta P_a^2 p_a, \tag{S23}$$

the Watson ($U^2$) statistic,

$$U^2 = k \sum_{a=a_0}^{a_m} [\Delta P_a - \langle \Delta P \rangle]^2 p_a, \tag{S24}$$

and the Anderson-Darling ($A^2$) statistic,

$$A^2 = k \sum_{a=a_0}^{a_m} \frac{\Delta P_a^2 p_a}{P_a(1 - P_a)}, \tag{S25}$$

where $\Delta P_a^2$ and $\langle \Delta P \rangle = \sum_{a=a_0}^{a_m} \Delta P_a p_a$ are, respectively, the square and average of the CDF difference between model and data [2].

The GOF test is as follows: Given the sample $\{a_i\}$ from an empirical ego network, we compute the MLE $\hat{\alpha}$ numerically from Eq. (S21), as well as the associated data statistics $D$, $W^2$, $U^2$, and $A^2$ from Eqs. (S22)-(S25), where the model CDF $P_a(t)$ is computed numerically from $p_a(t)$ in Eq. (S11) with $\alpha = \hat{\alpha}$ (and $t$, $a_0$, and $a_m$ are taken from the data sample). From the model $p_a(t)$ we generate

[2]For consistency, $\Delta P_{a_m} = 0$ and the last term in both $W^2$ and $A^2$ is set to zero.

| Dataset | $N$ | $n_\alpha$ | $n_\infty$ | $n_\emptyset$ | $N_\alpha$ | $n_{CA}$ | $n_{RN}$ |
|---|---|---|---|---|---|---|---|
| Mobile (call) | 5431921 | 0.70 | 0.06 | 0.23 | 3817319 | 0.95 | 0.05 |
| Mobile (sms) | 4233187 | 0.63 | 0.11 | 0.25 | 2687452 | 0.93 | 0.07 |
| Mobile (Wu 1) | 16050 | 0.42 | 0.25 | 0.33 | 6800 | 0.88 | 0.12 |
| Mobile (Wu 2) | 20534 | 0.38 | 0.27 | 0.35 | 7863 | 0.87 | 0.13 |
| Mobile (Wu 3) | 4215 | 0.36 | 0.33 | 0.31 | 1534 | 0.87 | 0.13 |
| Email (Enron) | 21984 | 0.39 | 0.34 | 0.27 | 8647 | 0.82 | 0.18 |
| Email (Kiel) | 9842 | 0.33 | 0.43 | 0.24 | 3266 | 0.83 | 0.17 |
| Email (Uni) | 2456 | 0.71 | 0.06 | 0.22 | 1746 | 0.95 | 0.05 |
| Email (EU) | 866 | 0.62 | 0.05 | 0.33 | 541 | 0.97 | 0.03 |
| Facebook | 31429 | 0.59 | 0.25 | 0.16 | 18689 | 0.83 | 0.17 |
| Messages | 20252 | 0.39 | 0.45 | 0.16 | 7814 | 0.68 | 0.32 |
| Dating | 16239 | 0.56 | 0.26 | 0.18 | 9135 | 0.85 | 0.15 |
| Forum | 4122 | 0.43 | 0.37 | 0.20 | 1762 | 0.66 | 0.34 |
| College | 1303 | 0.65 | 0.21 | 0.14 | 846 | 0.82 | 0.18 |
| CNS (call) | 285 | 0.34 | 0.29 | 0.37 | 97 | 0.85 | 0.15 |
| CNS (sms) | 347 | 0.54 | 0.07 | 0.39 | 188 | 0.99 | 0.01 |

**Table S3. Ego classes based on maximum likelihood estimation**. We classify the $N$ ego networks (with $t > a_0$) in each studied dataset into a fraction $n_\alpha = N_\alpha / N$ with statistically significant MLE $\hat{\alpha}$ (relative mean activity $t_r > 0$, p-value $p > 0.1$ according to statistic $D$, and $\hat{\alpha} < \alpha_b$ with $\alpha_b = 10^3$), a fraction $n_\infty = N_\infty / N$ with infinite $\hat{\alpha}$ [Eq. (S19) does not converge to zero below $\alpha_b$], and the remaining fraction $n_\emptyset = N_\emptyset / N$ with undefined $\hat{\alpha}$. The $N_\alpha$ egos with statistically significant $\hat{\alpha}$ are separated into a fraction $n_{RN} = N_{RN} / N_\alpha$ in the homogenous regime ($\beta < 1$), and a fraction $n_{CA} = N_{CA} / N_\alpha$ in the heterogeneous regime ($\beta > 1$).

$n_{\text{sim}} = 2500$ simulated activity samples $\{a_i\}_{\text{sim}}$. For each simulated sample, we find its own MLE $\hat{\alpha}_{\text{sim}}$ and the corresponding simulated statistics $D_{\text{sim}}$, $W^2_{\text{sim}}$, $U^2_{\text{sim}}$, and $A^2_{\text{sim}}$. Then, the fraction of simulated statistics $\bullet_{\text{sim}}$ larger than the data statistic $\bullet$ (i.e. $D_{\text{sim}} > D$ and so on) is the $p$-value $p_\bullet$ associated with the goodness-of-fit test, according to this particular test statistic. If the $p$-value is large enough ($p_\bullet > 0.1$ with 0.1 an arbitrary significance threshold), we do not rule out the hypothesis that our activity model emulates the empirical ego network. We aim at obtaining large $p$-values (rather than small), since we want to keep the assumption that the model is a good description of the observed data (rather than reject it).

We apply the MLE fitting procedure and GOF test described above to all $N$ ego networks of each communication dataset described in Section S1 and Table S1 (already filtered by the condition $t > a_0$ and thus with enough data on their heterogeneous communication patterns). Then we calculate the fraction $n_\bullet$ of egos that satisfy the condition $p_\bullet > 0.1$ according to statistic $\bullet$ (Table S2), that is, the fraction of egos well described by our model of alter activity. Values of $n_\bullet$ vary from 33% to 71% for $D$ and $A^2$, and slightly increase to the range 42–88% for $W^2$ and $U^2$. In this sense, the ability of our model to describe empirical data is robust to the measure of statistical significance, beyond small datasets for which both $W^2$ and $U^2$ are somewhat less restrictive. In large datasets like Mobile (call & sms), all four statistics imply that 63–70% of ego communication signatures are captured by the model. Given this similarity in the behaviour of test statistics, for the remaining of results (here and in the main text) we focus on the KS statistic $D$ and drop the notation $\bullet$ in the $p$-value.

The bootstrapped calculation of $p$-values allows us to separate egos into three categories depending of the properties of the MLE $\hat{\alpha}$ (Table S3):
• *Statistically significant* $\hat{\alpha}$. Out of $N$ egos with $t > a_0$ (so the empirical activity distribution is different from the initial condition of the model, see Section S3.1), in this category we only consider the $N_\alpha$ egos for which $p > 0.1$ (i.e. the GOF test does not rule out our model as a good description of the empirical

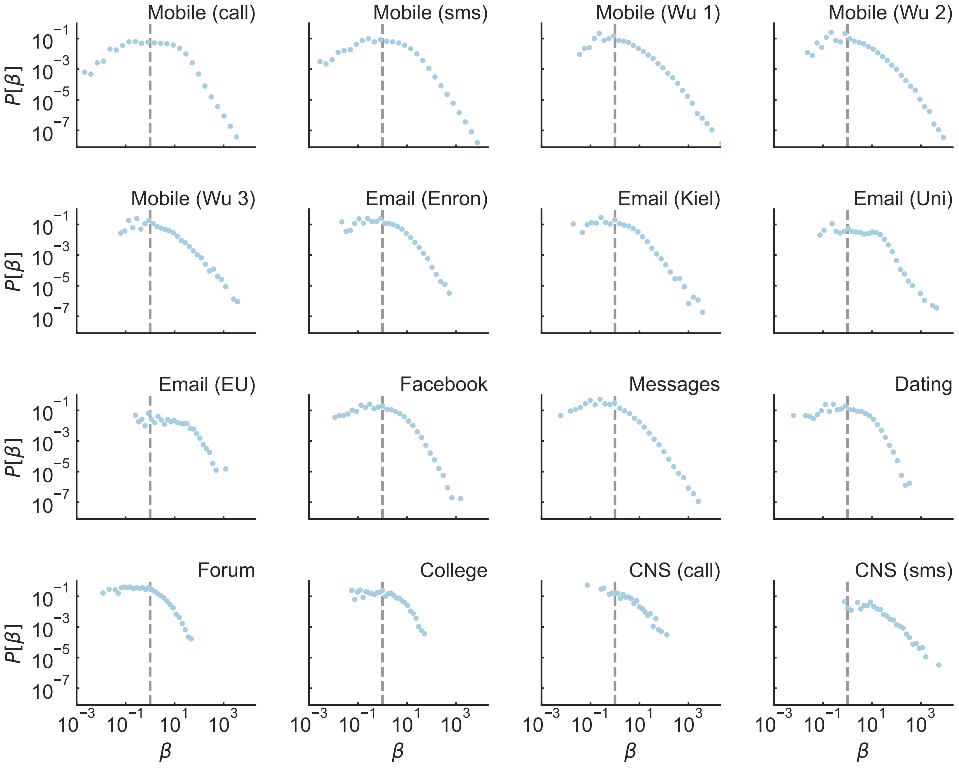


**Figure S11. Cumulative advantage vs. random choice in communication data.** Probability distribution $P[\beta]$ of estimated preferentiality parameter $\beta = t_r/\alpha_r$, fitted to the ego networks of several datasets via MLE (see Section S3). After only considering egos with $t_r > 0$ (i.e. $t > a_0$), $p > 0.1$ according to statistic $D$, and $\alpha < \alpha_b$ with $\alpha_b = 10^3$, ego networks have a similar distribution of $\beta$ values across datasets: a relatively small fraction of ego distribute contacts uniformly at random among their alters ($\beta < 1$), while most egos' contact activity is more heterogeneous and centered in a few of their alters ($\beta > 1$), exhibiting a broad distribution of preferentiality. In most systems, the distribution $P[\beta]$ peaks around the crossover $\beta = 1$ (dashed line).

ego network, according to the chosen statistic $D$). We also only take egos with $\hat{\alpha} < \alpha_b$, where $\alpha_b = 10^3$ is an arbitrary upper bound in the numerical calculation of MLEs via Eq. (S21), since the derivative of the log-likelihood in Eq. (S19) tends to 0 for $\alpha \to \infty$ (for fixed $a_0$ and $t$; see Fig. S10) and the numerical root search for Eq. (S21) can fail.

• *Infinite* $\hat{\alpha}$. From what remains, in this category we consider the $N_\infty$ egos for which the numerical search of the root of $F_\alpha - \ln(1+\beta)$ does not converge below the upper bound $\alpha_b$, and we assign to them the trivial solution of Eq. (S20), $\hat{\alpha} \to \infty$.

• *Undefined* $\hat{\alpha}$. The remaining $N_\emptyset$ egos have an undefined $\hat{\alpha}$ and are not considered further in our analysis of alter activity.

Table S3 shows the relative sizes of these classes for all datasets ($n_\alpha = N_\alpha/N$, $n_\infty = N_\infty/N$, and $n_\emptyset = N_\emptyset/N$, respectively, with $n_\alpha + n_\infty + n_\emptyset = 1$). Across all datasets, 33–71% of egos (with $t > a_0$) have communication patterns well captured by the model, while 5–45% are compatible with the trivial fit $\hat{\alpha} \to \infty$ and in this sense belong to the homogeneous regime of uniform alter activity. Overall, only a relatively small fraction of ego networks (14–39%) are not well emulated by the model, a figure that improves as we increase system size.

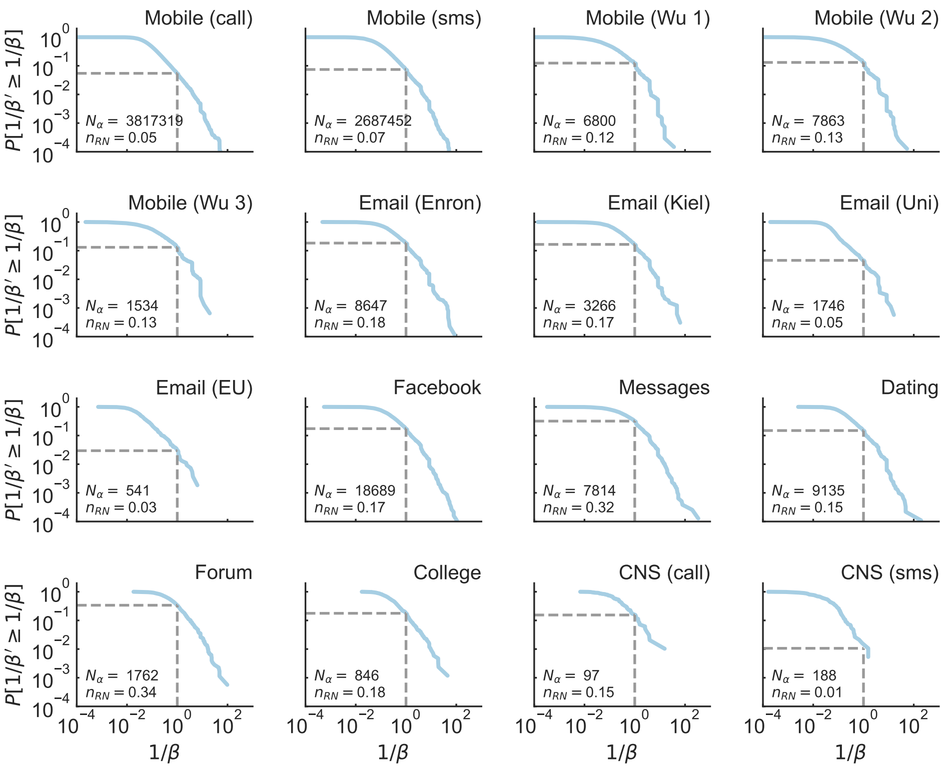


**Figure S12. Presence of cumulative advantage in communication data.** Complementary cumulative distribution function (CCDF) $P[1/\beta' \geq 1/\beta]$ of estimated rate $1/\beta = \alpha_r/t_r$, fitted to the ego networks of several datasets via MLE (see Section S3). After only considering egos with $t_r > 0$ (i.e. $t > a_0$), $p > 0.1$ according to statistic $D$, and $\alpha < \alpha_b$ with $\alpha_b = 10^3$, ego networks have a characteristic CCDF of the rate $1/\beta$ with similar shape across datasets: Out of $N_\alpha$ egos with statistically significant $\alpha$, a relatively small fraction $n_{RN}$ distribute contacts uniformly at random among their alters ($\beta < 1$), while most egos' contact activity is more heterogeneous and centered in a few of their alters ($\beta > 1$).

## S3.3 Activity regimes and persistence analysis in communication data

The distribution of statistically significant $\alpha$ values across egos has a similar shape in all datasets. We observe this in the distribution $P[\beta]$ of the estimated preferentiality parameter $\beta$ (Fig. S11), the CCDF $P[1/\beta' \geq 1/\beta]$ of the estimated rate $1/\beta = \alpha_r/t_r$ (Fig. S12), and in the associated scaling phase diagram in $(\alpha_r, t_r)$-space (Fig. S13; for comparison see Fig. S9) [3]. The way egos distribute communication events among alters lies in a spectrum: In the homogeneous regime of $\beta < 1$, a small fraction $n_{RN} = N_{RN}/N_\alpha$ of egos distribute activity among alters uniformly at random, with a Poissonian activity distribution asymptotically scaling like a Gaussian [see Section S2.1.2 and Eq. (S16)]. In the heterogeneous regime of $\beta > 1$, a larger fraction $n_{CA} = N_{CA}/N_\alpha$ of egos may concentrate their contacts in a just a few of their alters, so their activity is gamma-distributed with exponential scaling for given $\alpha_r$ [see Section S2.1.1 and Eq. (S14)]. Values of $n_{CA}$ and $n_{RN}$ are shown in Table S3 and Fig. S12, where $n_{CA} + n_{RN} = 1$. In most systems, the distribution $P[\beta]$ peaks around the crossover $\beta = 1$ separating scaling regimes, while the distribution of preferentiality values seems to be broader for heterogeneous egos than for homogeneous individuals (see Fig. S11). The two scaling regimes can also be distinguished from the CCDF $P[a' \geq a]$ of the number of alters having at least activity $a$, restricted to ego networks in each side of the crossover $\beta = 1$ (Fig. S14). When driven by cumulative advantage ($\beta > 1$), communication activity is larger and

[3] For simplicity, here and in the main text we drop the notation $\hat{\bullet}$ used for parameter estimates in Section S3.1 and Section S3.2.

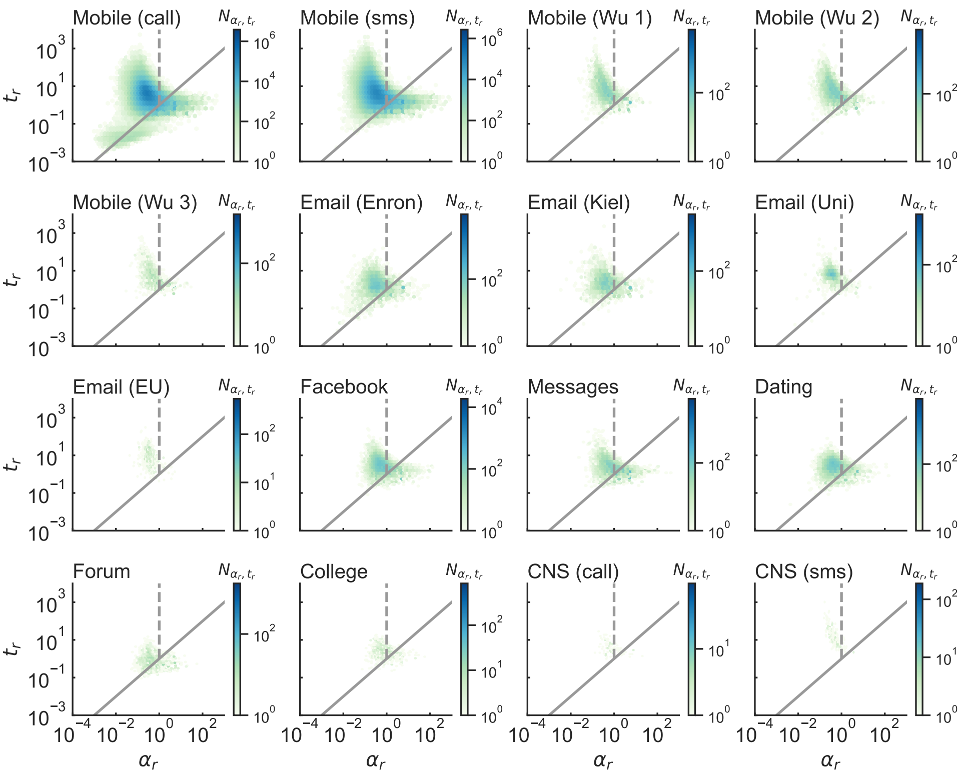


**Figure S13. Scaling phase diagram in communication data.** Hexbin histogram across datasets of number of egos $N_{\alpha_r,t_r}$ for given values of estimated parameter $\alpha_r = \alpha + a_0$ and relative mean activity $t_r = t - a_0$, with $a_0$ the minimum alter activity. The identity $\beta = t_r/\alpha_r = 1$ (continuous line) defines a crossover between regimes of scaling of the alter activity distribution $p_a(t)$. In the homogeneous regime ($\beta < 1$; bottom), a small fraction $n_{RN}$ of egos distribute contacts at random and $p_a$ scales like a Gaussian (see Table S3). In the heterogeneous regime ($\beta > 1$; top), $p_a$ approaches a gamma distribution and has exponential scaling. Most egos concentrate activity in a few alters ($\alpha_r < 1$; left of dashed line), while for a few egos, activity is distributed more uniformly across alters ($\alpha_r > 1$; right of dashed line).

more broadly distributed than in the homogeneous regime ($\beta < 1$), in consistence with the dispersion analysis of Section S1 (see Figs. S2-S3).

The presence of heterogeneous and homogeneous regimes of alter activity at the ego level, as well as the system-level similarity in the distribution of $\beta$ values across all datasets, are both features of human communication that seem to persist in time, regardless of potential changes in the identity of alters within ego networks (Fig. S15). To quantify this effect, we separate the observed period of activity of an ego network into two consecutive intervals with the same number of events ($I_1$ and $I_2$, see Fig. 1 in main text). We then independently estimate the preferentiality parameter for both the entire period ($\beta$) and for each of these two intervals ($\beta_1$ and $\beta_2$), leading to a preferentiality change $\Delta\beta = \beta_1 - \beta_2$. We also measure alter turnover as the Jaccard similarity coefficient $J = |A_1 \cap A_2|/|A_1 \cup A_2|$ between the sets of alters $A_1$ and $A_2$ in both intervals (with $J = 0$ implying totally different alters in $I_1$ and $I_2$, and $J = 1$ exactly the same alters across intervals) [39]. Fig. S15 shows that the relative preferentiality change $\Delta\beta/\beta$ stays close to zero regardless of alter turnover, somewhat trivially for $J \sim 1$ (since alters are anyway the same people when moving from $I_1$ to $I_2$), but remarkably also for $J \sim 0$. In other words, the individual way in which each ego allocates communication activity among alters (driven by cumulative advantage or by random alter choice) persists in time despite potentially large changes in the identity makeup of their social networks.

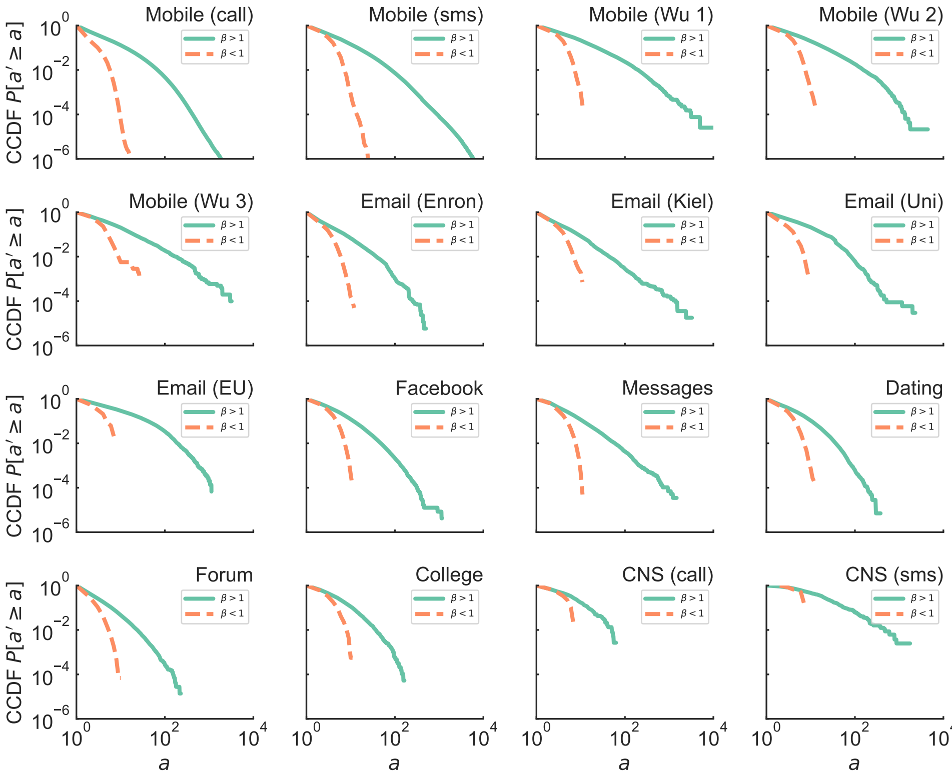


**Figure S14. Alter activity and preferentiality parameter.** Complementary cumulative distribution function (CCDF) $P[a' \geq a]$ of the number of alters having at least activity $a$, restricted to ego networks with estimated preferentiality $\beta$ in each side of the crossover ($\beta = 1$). Communication activity is larger and more broadly distributed when driven by cumulative advantage ($\beta > 1$, continuous lines), than by random choice ($\beta < 1$, dashed lines).

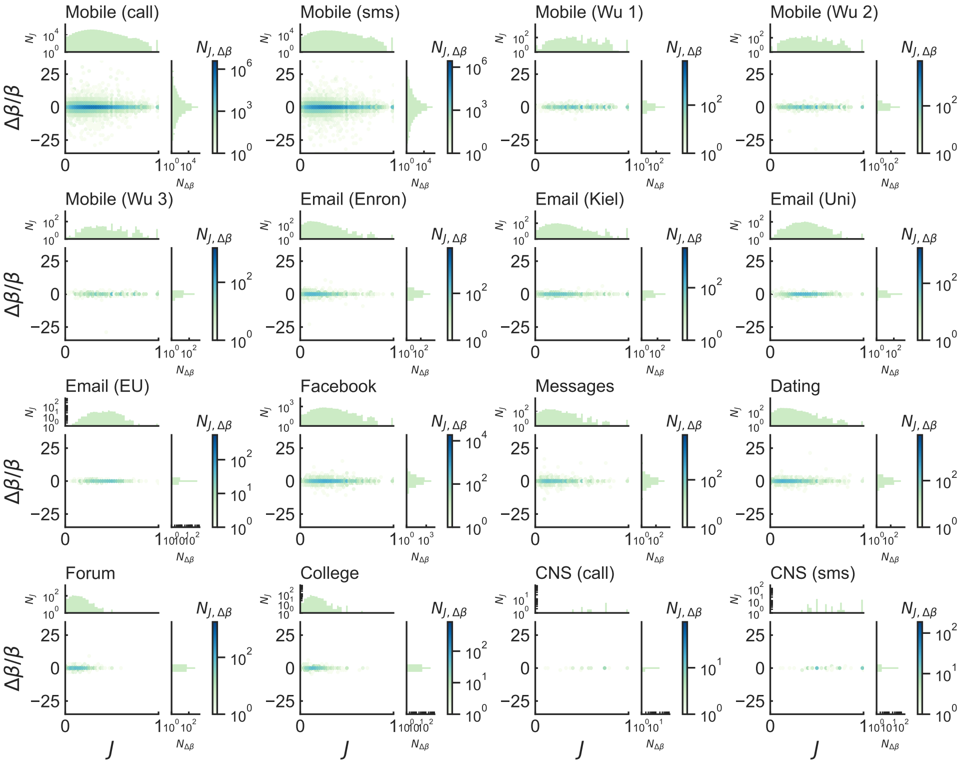


**Figure S15. Persistence of preferentiality in communication data.** Hexbin histogram across datasets of number $N_{J,\Delta\beta}$ of egos with given alter turnover $J$ and relative preferentiality change $\Delta\beta/\beta$. We estimate the preferentiality parameter in the whole observation period ($\beta$) as well as in two consecutive intervals of activity spanning the period ($\beta_1$ and $\beta_2$, respectively, with $\Delta\beta = \beta_1 - \beta_2$). We also show marginal number distributions of turnover ($N_J$) and relative preferentiality change ($N_{\Delta\beta}$). Social signatures are persistent in time at the level of individuals, regardless of alter turnover.

## Supplementary References


[1] J.-P. Onnela, J. Saramäki, J. Hyvönen, G. Szabó, M. A. De Menezes, K. Kaski, A.-L. Barabási, and J. Kertész, "Analysis of a large-scale weighted network of one-to-one human communication," *New J. Phys.*, vol. 9, no. 6, p. 179, 2007.

[2] J.-P. Onnela, J. Saramäki, J. Hyvönen, G. Szabó, D. Lazer, K. Kaski, J. Kertész, and A.-L. Barabási, "Structure and tie strengths in mobile communication networks," *Proc. Nat. Acad. Sci. USA*, vol. 104, no. 18, pp. 7332–7336, 2007.

[3] M. Karsai, M. Kivelä, R. K. Pan, K. Kaski, J. Kertész, A.-L. Barabási, and J. Saramäki, "Small but slow world: How network topology and burstiness slow down spreading," *Phys. Rev. E*, vol. 83, no. 2, p. 025102, 2011.

[4] M. Kivelä, R. K. Pan, K. Kaski, J. Kertész, J. Saramäki, and M. Karsai, "Multiscale analysis of spreading in a large communication network," *J. Stat. Mech.*, vol. 2012, no. 03, p. P03005, 2012.

[5] L. Kovanen, K. Kaski, J. Kertész, and J. Saramäki, "Temporal motifs reveal homophily, gender-specific patterns, and group talk in call sequences," *Proc. Nat. Acad. Sci. USA*, vol. 110, no. 45, pp. 18070–18075, 2013.

[6] S. Unicomb, G. Iñiguez, and M. Karsai, "Threshold driven contagion on weighted networks," *Sci. Rep.*, vol. 8, no. 1, pp. 1–10, 2018.

[7] S. Heydari, S. G. Roberts, R. I. M. Dunbar, and J. Saramäki, "Multichannel social signatures and persistent features of ego networks," *Appl. Netw. Sci.*, vol. 3, no. 1, p. 8, 2018.

[8] Y. Wu, C. Zhou, J. Xiao, J. Kurths, and H. J. Schellnhuber, "Evidence for a bimodal distribution in human communication," *Proc. Nat. Acad. Sci. USA*, vol. 107, no. 44, pp. 18803–18808, 2010.

[9] B. Klimt and Y. Yang, "The Enron corpus: A new dataset for email classification research," in *Proc. Eur. Conf. Machine Learning (ECML)*, pp. 217–226, Springer, 2004.

[10] J. Kunegis, "KONECT: The Koblenz network collection," in *Proc. Int. Conf. on World Wide Web Companion*, pp. 1343–1350, 2013.

[11] H. Ebel, L.-I. Mielsch, and S. Bornholdt, "Scale-free topology of e-mail networks," *Phys. Rev. E*, vol. 66, no. 3, p. 035103, 2002.

[12] J. Saramäki and P. Holme, "Exploring temporal networks with greedy walks," *Eur. Phys. J. B*, vol. 88, no. 12, p. 334, 2015.

[13] J.-P. Eckmann, E. Moses, and D. Sergi, "Entropy of dialogues creates coherent structures in e-mail traffic," *Proc. Nat. Acad. Sci. USA*, vol. 101, no. 40, pp. 14333–14337, 2004.

[14] J. Leskovec, J. Kleinberg, and C. Faloutsos, "Graph evolution: Densification and shrinking diameters," *ACM Trans. Knowl. Disc. Data*, vol. 1, no. 1, p. 2, 2007.

[15] A. Paranjape, A. R. Benson, and J. Leskovec, "Motifs in temporal networks," in *Proc. 10th ACM Int. Conf. on Web Search and Data Mining*, pp. 601–610, 2017.

[16] B. Viswanath, A. Mislove, M. Cha, and K. P. Gummadi, "On the evolution of user interaction in Facebook," in *Proceedings of the 2nd ACM workshop on Online social networks*, pp. 37–42, 2009.

[17] A. Said, E. W. De Luca, and S. Albayrak, "How social relationships affect user similarities," in *Proc. of the 2010 workshop on social recommender systems*, pp. 1–4, 2010.

[18] F. Karimi, V. C. Ramenzoni, and P. Holme, "Structural differences between open and direct communication in an online community," *Phys. A*, vol. 414, pp. 263–273, 2014.

[19] P. Holme, C. R. Edling, and F. Liljeros, "Structure and time evolution of an internet dating community," *Soc. Net.*, vol. 26, no. 2, pp. 155–174, 2004.

[20] T. Opsahl and P. Panzarasa, "Clustering in weighted networks," *Soc. Net.*, vol. 31, no. 2, pp. 155–163, 2009.

[21] P. Panzarasa, T. Opsahl, and K. M. Carley, "Patterns and dynamics of users' behavior and interaction: Network analysis of an online community," *J. Am. Soc. Inf. Sci. Tec.*, vol. 60, no. 5, pp. 911–932, 2009.

[22] A. Stopczynski, V. Sekara, P. Sapiezynski, A. Cuttone, M. M. Madsen, J. E. Larsen, and S. Lehmann, "Measuring large-scale social networks with high resolution," *PloS ONE*, vol. 9, no. 4, p. e95978, 2014.

[23] P. Sapiezynski, A. Stopczynski, D. D. Lassen, and S. Lehmann, "Interaction data from the Copenhagen Networks Study," *Sci. Data*, vol. 6, no. 1, pp. 1–10, 2019.

[24] CALO Project, "Enron email dataset," 2015. `https://www.cs.cmu.edu/~enron/`.

[25] P. Sapiezynski, A. Stopczynski, D. D. Lassen, and S. L. Jørgensen, "The Copenhagen Networks Study interaction data," *figshare*, 2019. URL: `https://doi.org/10.6084/m9.figshare.7267433`.

[26] K.-I. Goh and A.-L. Barabási, "Burstiness and memory in complex systems," *Europhys. Lett.*, vol. 81, no. 4, p. 48002, 2008.

[27] D. Price, "Networks of scientific papers," *Science*, pp. 510–515, 1965.

[28] D. Price, "A general theory of bibliometric and other cumulative advantage processes," *J Am. Soc. Inform. Sci.*, vol. 27, no. 5, pp. 292–306, 1976.

[29] M. Newman, *Networks*. Oxford University Press, 2018.

[30] A. Clauset, C. R. Shalizi, and M. E. J. Newman, "Power-law distributions in empirical data," *SIAM Rev.*, vol. 51, no. 4, pp. 661–703, 2009.

[31] J. A. Morales, S. Sánchez, J. Flores, C. Pineda, C. Gershenson, G. Cocho, J. Zizumbo, R. F. Rodríguez, and G. Iñiguez, "Generic temporal features of performance rankings in sports and games," *EPJ Data Sci.*, vol. 5, no. 1, p. 33, 2016.

[32] I. Voitalov, P. van der Hoorn, R. van der Hofstad, and D. Krioukov, "Scale-free networks well done," *Phys. Rev. Research*, vol. 1, no. 3, p. 033034, 2019.

[33] M. A. Stephens, "EDF statistics for goodness of fit and some comparisons," *J. Am. Stat. Assoc.*, vol. 69, no. 347, pp. 730–737, 1974.

[34] W. H. Press, S. A. Teukolsky, W. T. Vetterling, and B. P. Flannery, "Numerical recipes in C," 1988.

[35] T. W. Anderson, "On the distribution of the two-sample Cramér–von Mises criterion," *Ann. Math. Stat.*, pp. 1148–1159, 1962.

[36] V. Choulakian, R. A. Lockhart, and M. A. Stephens, "Cramér-von Mises statistics for discrete distributions," *Can. J. Stat.*, pp. 125–137, 1994.

[37] S. Csörgő and J. J. Faraway, "The exact and asymptotic distributions of Cramér-von Mises statistics," *J. R. Stat. Soc. B*, vol. 58, no. 1, pp. 221–234, 1996.

[38] R. A. Lockhart, J. J. Spinelli, and M. A. Stephens, "Cramér–von Mises statistics for discrete distributions with unknown parameters," *Can. J. Stat.*, pp. 125–133, 2007.

[39] J. Saramäki, E. A. Leicht, E. López, S. G. B. Roberts, F. Reed-Tsochas, and R. I. M. Dunbar, "Persistence of social signatures in human communication," *Proc. Nat. Acad. Sci. USA*, vol. 111, no. 3, pp. 942–947, 2014.
